# Proteomic insight into fruit set of cucumber (*Cucumis sativus* L.) suggests the cues of hormone-independent parthenocarpy

**DOI:** 10.1186/s12864-017-4290-5

**Published:** 2017-11-22

**Authors:** Ji Li, Jian Xu, Qin-Wei Guo, Zhe Wu, Ting Zhang, Kai-Jing Zhang, Chun-yan Cheng, Pin-yu Zhu, Qun-Feng Lou, Jin-Feng Chen

**Affiliations:** State Key Laboratory of Crop Genetics and Germplasm Enhancement, Nanjing Agricultural University, Nanjing, 210095 China

**Keywords:** Cucumber (*Cucumis sativus* L.), Parthenocarpy, Proteome, iTRAQ, Hormone dependent/independent

## Abstract

**Background:**

Parthenocarpy is an excellent agronomic trait that enables crops to set fruit in the absence of pollination and fertilization, and therefore to produce seedless fruit. Although parthenocarpy is widely recognized as a hormone-dependent process, hormone-insensitive parthenocarpy can also be observed in cucumber; however, its mechanism is poorly understood. To improve the global understanding of parthenocarpy and address the hormone-insensitive parthenocarpy shown in cucumber, we conducted a physiological and proteomic analysis of differently developed fruits.

**Results:**

Physiological analysis indicated that the natural hormone-insensitive parthenocarpy of ‘EC1’ has broad hormone-inhibitor resistance, and the endogenous hormones in the natural parthenocarpy (NP) fruits were stable and relatively lower than those of the non-parthenocarpic cultivar ‘8419 s-1.’ Based on the iTRAQ technique, 683 fruit developmental proteins were identified from NP, cytokinin-induced parthenocarpic (CP), pollinated and unpollinated fruits. Gene Ontology (GO) analysis showed that proteins detected from both set and aborted fruits were involved in similar biological processes, such as cell growth, the cell cycle, cell death and communication. Kyoto Encyclopedia of Genes and Genomes (KEGG) analysis revealed that ‘protein synthesis’ was the major biological process that differed between fruit set and fruit abortion. Clustering analysis revealed that different protein expression patterns were involved in CP and NP fruits. Forty-one parthenocarpy-specialized DEPs (differentially expressed proteins) were screened and divided into two distinctive groups: NP-specialized proteins and CP-specialized proteins. Furthermore, qRT-PCR and western blot analysis indicated that NP-specialized proteins showed hormone- or hormone-inhibitor insensitive expression patterns in both ovaries and seedlings.

**Conclusions:**

In this study, the global molecular regulation of fruit development in cucumber was revealed at the protein level. Physiological and proteomic comparisons indicated the presence of hormone-independent parthenocarpy and suppression of fruit abortion in cucumber. The proteomic analysis suggested that hormone-independent parthenocarpy is regulated by hormone-insensitive proteins such as the NP-specialized proteins. Moreover, the regulation of fruit abortion suppression may be closely related to protein synthesis pathways.

**Electronic supplementary material:**

The online version of this article (10.1186/s12864-017-4290-5) contains supplementary material, which is available to authorized users.

## Background

Parthenocarpy is considered the most cost-effective solution for improving the fruit set rate when pollination or fertilization is suppressed by sub-optimum growth conditions, such as low temperature, weak light intensity or facility environments, and ensuring yields of vegetable and fruit crops that are self-sterile or gynoecious. Moreover, seedless fruits produced by parthenocarpy have a better texture, appearance and shelf life [1] and avoid yield loss caused by seed development [2–4].

Parthenocarpy is a widely recognized hormone-dependent biological process. Independent evidence indicates that auxins play a vital role in parthenocarpic fruit set. The genes linking the auxin signal transduction pathway to fruit set have been identified in recent decades. The involvement of IAA9, a member of the tomato Aux/IAA gene family of transcriptional regulators, was confirmed in tomato fruit set. Auxin dose-response assays showed that the down-regulation of IAA9 led to auxin hypersensitivity and resulted in parthenocarpy [5]. Another auxin signaling component involved in fruit set is ARF8, which was identified as a candidate gene for two parthenocarpy QTLs in tomato [6]. Based on the findings of Goetz et al. [7], a model was proposed for the mechanism of parthenocarpic induction. According to this model, ARF8 forms an inhibitory complex together with an AUX/IAA protein, possibly IAA9, to repress the transcription of the auxin response genes and consequently induce parthenocarpy. Wittwer et al. [8] showed that a second class of hormone, gibberellins (GAs), could also stimulate parthenocarpic fruit set. The only known gibberellin signaling component shown to be involved in fruit set is *DELLA*. The reduction in *SlDELLA* mRNA levels induces the formation of parthenocarpic tomato fruit [9]. Null and loss-of-function recessive mutations in the *DELLA* genes of *Arabidopsis* provoke a constitutive GA-response phenotype, including parthenocarpy [10]. Besides Auxin and GAs, Cytokinin (CK) is also involved in parthenocarpy, which accumulates to high levels in ovaries during fruit set [11–15]. Recent studies suggested that CKs may induce parthenocarpy partially through modulation of IAA and GA metabolisms [16–18]. Ethylene and abscisic acid (ABA) also play important roles in the regulation of fruit set and development. Ethylene is likely involved in the fruit set program by functioning coordinately with auxin [19–21]. ABA may acts as an antagonist of GA or auxin to induce and maintain the dormant state of ovaries, likely by repressing their transition to fruit [20]. These studies demonstrate the complicated and confusing relationships among hormone responses during fruit set. However, the key integrating molecular players remain largely undiscovered, and a global understanding of the mechanisms underlying parthenocarpy has yet to be attained.

Genetic studies have suggested that a majority of natural parthenocarpic properties in crops are quantitative traits regulated by both genetic and environmental factors [6, 22–26]. Photoperiod, temperature, light intensity and nutritional conditions have considerable influences on parthenocarpy [27–29]. A hypothesis proposed in the 1930s suggested that plant developmental responses to environmental stimuli were due to the spatiotemporal variations in phytohormone synthesis and transport [30, 31]. Studies have suggested that short-daylight conditions could enhance parthenocarpy by increasing the activity of auxin, while high temperatures suppressed the parthenocarpy rate by inhibiting the synthesis of auxin and gibberellin in the ovary of cucumber [32, 33]. Kim et al. [34] also found that ovaries had twice the auxin content at 15 °C than that at 25 °C, resulting in a higher rate of parthenocarpy in cucumber. Anyhow, the agricultural application of parthenocarpy was limited by its environmental sensitivity. In practice, the excessive application of exogenous hormones was often used to overwhelm the environmental effects on hormone synthesis, thereby inducing environmentally stable parthenocarpy.

Cucumber (*Cucumis sativus* L.) is emerging as a model for plants in the *Cucurbitaceae* family because of its small and fully sequenced genome (2n = 2x = 14, 367 Mb genome) [35]. The mechanism for sex determination, vascular system development, and typical pepo fruit are also well documented in cucumber. The rich parthenocarpic germplasm of cucumber offers an opportunity to investigate the coordination and communication of hormone signals and genes during parthenocarpic fruit set. Genetic studies of parthenocarpy in cucumber started in 1930s. Early studies suggested that parthenocarpy in cucumber is controlled by single genes [36–40]. While most recent studies confirmed that inheritance of parthenocarpy in cucumber is consistent with characteristics of quantitative traits [25, 26, 41–43]. Although cucumber is rich in parthenocarpic germplasm resources e.g. main branch specialized/lateral branch specialized parthenocarpy, temperature/photoperiods sensitive parthenocarpy and parthenocarpy with accelerated ovary expansion before anthesis, the agricultural application of parthenocarpic cucumber was limited by their environmental sensitivity [43]. A serial of studies indicated that stable parthenocarpy of cucumber can be induced by auxin or auxin transport inhibitors [34, 44–48]. Meanwhile artificially increasing of endogenous auxin in the ovary by introducing the DefH9-iaaM auxin-synthesizing gene into cucumber might also stimulate parthenocarpy [49]. Besides auxin, application of other hormones such as cytokinins, gibberenllins and brassinosteroids (BRs) could also promote parthenocarpy in cucumber [50, 51]. However, it reported that auxin and GAs had less potential to induce parthenocarpic fruit growth than CKs in cucumber [34, 51, 52]. Therefore, in practice application of exogenous cytokinin, particularly CPPU (N-(2-chloro-4-pyridyl)-N′-phenyl urea, a type of synthetic cytokinin), to induce parthenocarpy is widely used in cucumber production.

In previous studies, an excellent parthenocarpic cucumber cultivar, ‘EC1,’ was found, which showed environmentally stable parthenocarpy under different culture conditions [25, 43]. Previous transcriptome studies have demonstrated that the natural parthenocarpy (NP) of ‘EC1’ has many different aspects compared with cytokinin-induced parthenocarpy (CP) at the mRNA level [53]. However, mRNA levels are not always in accordance with protein activity. To improve the global understanding of parthenocarpy and address the environmental stability of parthenocarpy in cucumber, we conducted a physiological analysis and an iTRAQ (isobaric tags for relative and absolute quantitation)-based proteomic analysis in the natural parthenocarpic fruits of ‘EC1’ and cytokinin-induced parthenocarpic fruits of ‘8419 s-1’ (a non-parthenocarpic variety).

## Results

### Physiological comparison of the parthenocarpic and non-parthenocarpic cucumber cultivars

**Fig. 1:**
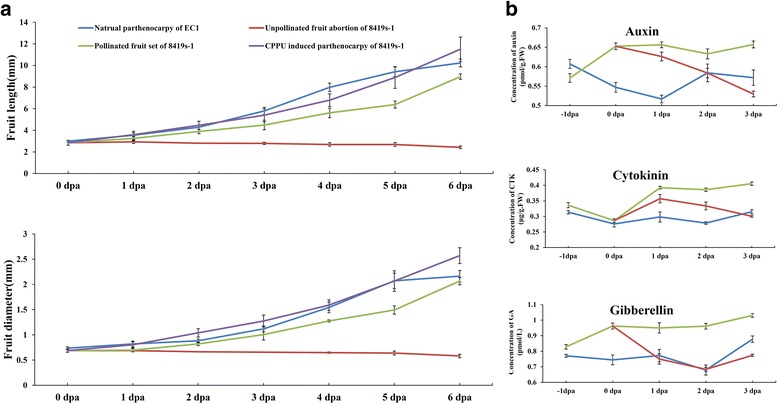
Growth curve and endogenous hormone analysis of different cucumber fruits. **a** The length and diameter of natural parthenocarpic fruits of ‘EC1,’ CPPU-induced parthenocarpic ‘8419 s-1’ fruits, and the pollinated and unpollinated fruits of ‘8419 s-1’ were measured from 0 dpa (days post-anthesis) to 6 dpa. Each value represents the mean ± SE (*n* = 30). **b** The concentrations of auxins, cytokinins and gibberellins in the natural parthenocarpic fruits of ‘EC1’ and pollinated/unpollinated fruit of ‘8419 s-1’ at −1 dpa to 3 dpa (analyzed by ELISA). The results are presented as mean ± SE of three repeated sample pools (*n* = 10) with three technical replicates

Experiments were conducted to investigate the physiological differences between parthenocarpic cultivar ‘EC1’ and non-parthenocarpic cultivar ‘8419 s-1’ during natural/cytokinin-induced parthenocarpy, pollinated fruit set and unpollinated fruit abortion. The detail information of the two cultivars was described in Material and Methods section. The longitudinal and radial growth of the natural parthenocarpic fruits of ‘EC1’ and CPPU -induced parthenocarpy of ‘8419 s-1’, pollinated and unpollinated fruits of ‘8419 s-1’ were measured (Fig. 1a). Our results showed that the length and diameter of the parthenocarpic and pollinated fruits linearly increased from 0 to 6 dpa (days post-anthesis), and the natural and CPPU-induced parthenocarpic fruits showed similar growth curves, wherein the fruit size was generally larger than the pollinated fruits. In contrast, the growth of the unpollinated fruits of ‘8419 s-1’ was blocked, and the length and diameter of the abortive fruits also decreased slightly.

Kim et al. [34] suggested that genetic factor for parthenocarpy in cucumber may be associated with high content of IAA in the ovaries at anthesis. In this study, endogenous auxins, cytokinins, and gibberellins were analyzed in the cucumber fruits noted above (Fig. 1b). The induction of both naturally occurring and hormone induced parthenocarpy is attributed to the presence of sufficient phytohormones in the ovaries [54–58]. However, parthenocarpic fruits of ‘EC1’ had relatively low and stable hormone levels compared with the fruits of ‘8419 s-1.’ Moreover, the auxin and gibberellin concentrations also decreased unexpectedly during the natural parthenocarpic fruit set of ‘EC1’ (Fig. 1b).

For a further comparison of the fruit developmental differences between ‘EC1’ and ‘8419 s-1,’ we conducted ovary treatment experiments. The ovaries of ‘EC1’ and ‘8419 s-1’ were treated with hormones, hormone inhibitors and pollen separately at anthesis. The weight, length and diameter measurement of the treated ovaries was conducted at 4dpa to reveal different phytohormone responses between ‘EC1’ and ‘8419 s-1’. In cucumber, etiolation of ovary tips is the principal identifying symbol to identify whether the fruits are set or aborted since 2 dpa. It was showed that the ovaries with etiolated tips did not grow or even wilt by comparing with the 0 dpa ovaries (Table 1). The non-etiolated tip phenotypes and growth of ovaries suggested that parthenocarpy of ‘8419 s-1’ could be induced by all of the exogenous hormones, including NAA, CPPU, GA_3_ and EBR; however, NAA, GA3 and EBR exerted weak effects on fruit growth, causing the treated ovaries to grow slightly in length and remain in a dormant-like state (Table 1, Additional file 1: Figure S1). Marcelis et al. [59] suggested that cell division in fruit of cucumber occurs about a week after anthesis whereas cell size increases markedly only after cell division begins to decline. Fruit size increases because of increase in both the number and size of cells. Therefore we speculated that NAA, GA3 and EBR might be primarily involved in cell division, rather than cell expansion during fruit development of cucumber, thus ovaries treated by these hormones are smaller than pollinated and CCPU-treated fruits. On the other hand, the weak effect of NAA, GA3 and EBR might be due to the low hormone concentration used in this study. Fu et al. [51] showed that 0.2 μM (20 mg/L) of EBR led to high efficiency of fruit growth in cucumber. But in this study we found 10 mg/L of EBR showed weak effect on fruit expansion. However, half concentration of CPPU (50 mg/L) referred to Fu’s method still lead to strong effect on fruit set and growth.

**Table 1:** The measurement of ovary weight, length and diameter after treated by pollen, exogenous hormones and hormone inhibitors

		0 dpa ovary	Unpollination ^b^	Pollination I^c^	Pollination II	NAA	CPPU	GA_3_	EBR	Hormone Mix^d^
Ovaries of 8419 s-1	Weight (g)	0.75 ± 0.14 D^a^	0.66 ± 0.15 D	8.39 ± 1.4 B	5.05 ± 0.94 C	1.01 ± 0.12 D	12.81 ± 1.25 A	1.01 ± 0.17 D	0.86 ± 0.21 D	13.04 ± 3.14 A
	Length (mm)	28.4 ± 2.4 E	27.60 ± 3.3 E	69.33 ± 4.25 B	58.91 ± 3.96 C	34.06 ± 1.91 D	77.76 ± 5.67 A	33.31 ± 2.59 D	31.31 ± 3.1 DE	81.43 ± 6.77 A
	Diameter (mm)	6.86 ± 0.33 D	6.30 ± 0.48 D	13.46 ± 1.38 B	11.07 ± 2.8 C	6.96 ± 0.7 D	15.87 ± 1.9 A	6.98 ± 0.58 D	6.73 ± 0.33 D	14.99 ± 2.3 A
	P/A/T^e^		0/30/30	–	–	31/0/31	30/0/30	36/0/36	34/0/34	35/0/35
		TIBA	Lovastatin	Uniconazole	Brz	Inhibitor Mix ^g^				
Pollinated ovaries of 8419 s-1^f^	Weight (g)	0.67 ± 0.06 D	0.67 ± 0.05 D	0.69 ± 0.04 D	0.68 ± 0.03 D	0.62 ± 0.08 D				
	Length (mm)	25.7 ± 0.55 F	26.8 ± 1.11 E	25.9 ± 1.59 E	26.9 ± 1.27 E	26.5 ± 1.40 E				
	Diameter (mm)	5.96 ± 0.24 F	5.84 ± 0.05 F	5.70 ± 0.35 F	5.69 ± 0.31 F	6.07 ± 0.19 F				
	P/A/T	0/35/35	0/29/29	0/30/30	0/32/32	0/30/30				
		0 dpa ovary	Unpollination	Pollination I		TIBA	Lovastatin	Uniconazole	Brz	Inhibitor Mix
Ovaries of EC1	Weight (g)	0.83 ± 0.07 E	8.09 ± 1.51 A	2.64 ± 0.49 B		1.56 ± 0.23 CD	1.22 ± 0.37 CDE	0.93 ± 0.13 E	1.76 ± 0.38 C	1.11 ± 0.2 DE
	Length (mm)	30.6 ± 3.1 F	58.70 ± 2.74 A	45.27 ± 1.51 B		34.82 ± 1.74 D	34.52 ± 0.58 D	33.89 ± 0.55 DE	38.06 ± 1.33 C	32.3 ± 2.53 EF
	Diameter (mm)	6.87 ± 0.27 E	10.58 ± 1.11 A	8.39 ± 1.47 BC		7.20 ± 0.38 DE	7.17 ± 0.78 DE	8.81 ± 1.33 B	9.16 ± 1.35 B	7.84 ± 0.87D
	P/A/T		30/0/30	–		30/0/30	30/0/30	30/0/30	30/0/30	37/0/37

The treatments were conducted at the anthesis day (0 dpa), and the measurements were conducted at 4 dpa. Means (±SE) of three independent experiments were calculated

^a^Letters indicate differences between the treated ovaries with statistical significance at P ≤ 0.05 (t-test). The same letter means not significantly different; different letters means significantly different

^b^Underline words means the treatment can induce etiolation of ovary tips at 4dpa and lead to fruit abortion

^c^‘Pollination I’ means hand pollination with active pollens; ‘Pollination II’ means hand pollination with irradiated pollens (γ-ray irradiation at a dose of 200 Gy)

^d^Mixed solution of NAA (50 mg/L), CPPU (50 mg/L), GA3 (50 mg/L) and EBR (10 mg/L)

^e^P/A/T: number of parthenocarpic ovaries/number of abortive ovaries/total treated ovaries; etiolation phenotype of ovary tips is the principal identifying symbol to identify whether the fruits are set or aborted since 2dpa

^f^Ovaries of 8419 s-1 were pollinated at 0 dpa, the hormone inhibitor treatments were conducted 3 h after pollination

^g^Mixed solution of TIBA (50 mg/L), Lovastatin (50 mg/L), uniconazole (50 mg/L) and Brz (10 mg/L)

The strong effect of CPPU on fruit development of cucumber was observed and documented in many other reports [34, 51, 52]. Similar observation was also found in many other species such as watermelon, apple, kiwifruit and blueberry [60–63]. Early fruit development generally consists of three stages: fruit formation, cell division and cell expansion [13]. The growth of cucumber fruit size is often mirrored by the increase in cell number and size [64]. Previous cytological observation showed that the CPPU-treated cucumber fruits was initiated with an increase of cell numbers in the pericarp and placenta tissues, and the size of pericarp cells were bigger than natural parthenocarpic fruits, although the number of cell layers was similar [53]. The findings suggested that CPPU might be involved in both processes of cell division and cell expansion. In addition, fruit growth is tightly related to the availability of carbohydrate, because fruit is a very strong metabolic sink. Many studies confirmed that CKs was demonstrated to regulate carbohydrate allocation in fruit [65, 66]. We thought that maybe another reason why CPPU induced parthenocarpic fruit was consistently bigger than the fruit induced by other PRGs.

Martínez et al. [21] have demonstrated that the inhibition of ethylene response (STS treatment) is sufficient to induce the set and early development of the fruit in absence of pollination in both the parthenocarpic and the non-parthenocarpic cultivar of zucchini squash. Coincidentally, it was showed that STS has stimulated parthenocarpy in the three non-parthenocarpic cucumber cultivars; however it had no effect on fruit development of parthenocarpic cultivar ‘EC1’ (Additional file 2: Table S6). Besides, diameter, length and weight of ethephon treated fruit of ‘EC1’ showed no significant differences to the natural parthenocarpic fruits (Additional file 2: Table S6). It indicated that neither ethephon nor STS (ethylene response inhibitor) could affect parthenocarpy of EC. Interestingly, the ethephon treated ovaries of non-parthenocarpic cucumbers displayed more severe atrophy of ovaries by comparing with their unpollinated ovaries (Additional file 2: Table S6), suggested that the fruit abortion of the non-parthenocarpic cultivars maybe accelerated by ethephon. Irradiated pollen treatment could promote stenospermocarpy in 8419 s-1, but the stenospermocarpic ovaries were much smaller than the active pollen-treated ovaries, implying that seed set may be essential for the fruit growth of non-parthenocarpic varieties. Interestingly, the seedless fruit of ‘EC1’ formed by parthenocarpy was much larger than its pollinated fruit. The pollination fruits of ‘8419 s-1’ were blocked by either a hormone inhibitor mixture or individual hormone inhibitors (Table 1). Etiolation was observed in the pollen and hormone inhibitor co-treated ovaries. In contrast, the natural parthenocarpic fruit set of ‘EC1’ could not be blocked by hormone inhibitors, but the growth of the ovaries was suppressed (Table 1).

### iTRAQ-based proteomic study of differently developed cucumber fruits

Early fruit development generally consists of three stages: fruit formation, cell division and cell expansion [13]. The earliest stage, in which the ovary is aborted or allowed to proceed with fruit development, is referred to as fruit set. In this study, we focused on the fruit set stage in cucumber, which is 0 to 2 days post-anthesis [53, 59, 64, 67, 68]. The natural parthenocarpic fruits of ‘EC1,’ cytokinin-induced parthenocarpic fruits of ‘8419 s-1’, the pollinated and unpollinated fruits of ‘8419 s-1’ were investigated (described in the M&M section). The proteomes of these differently developed fruits were analyzed by iTRAQ with two technical replicates per sample. The strategy for analysis is shown in Additional file 3: Figure S2.

**Fig. 2:**
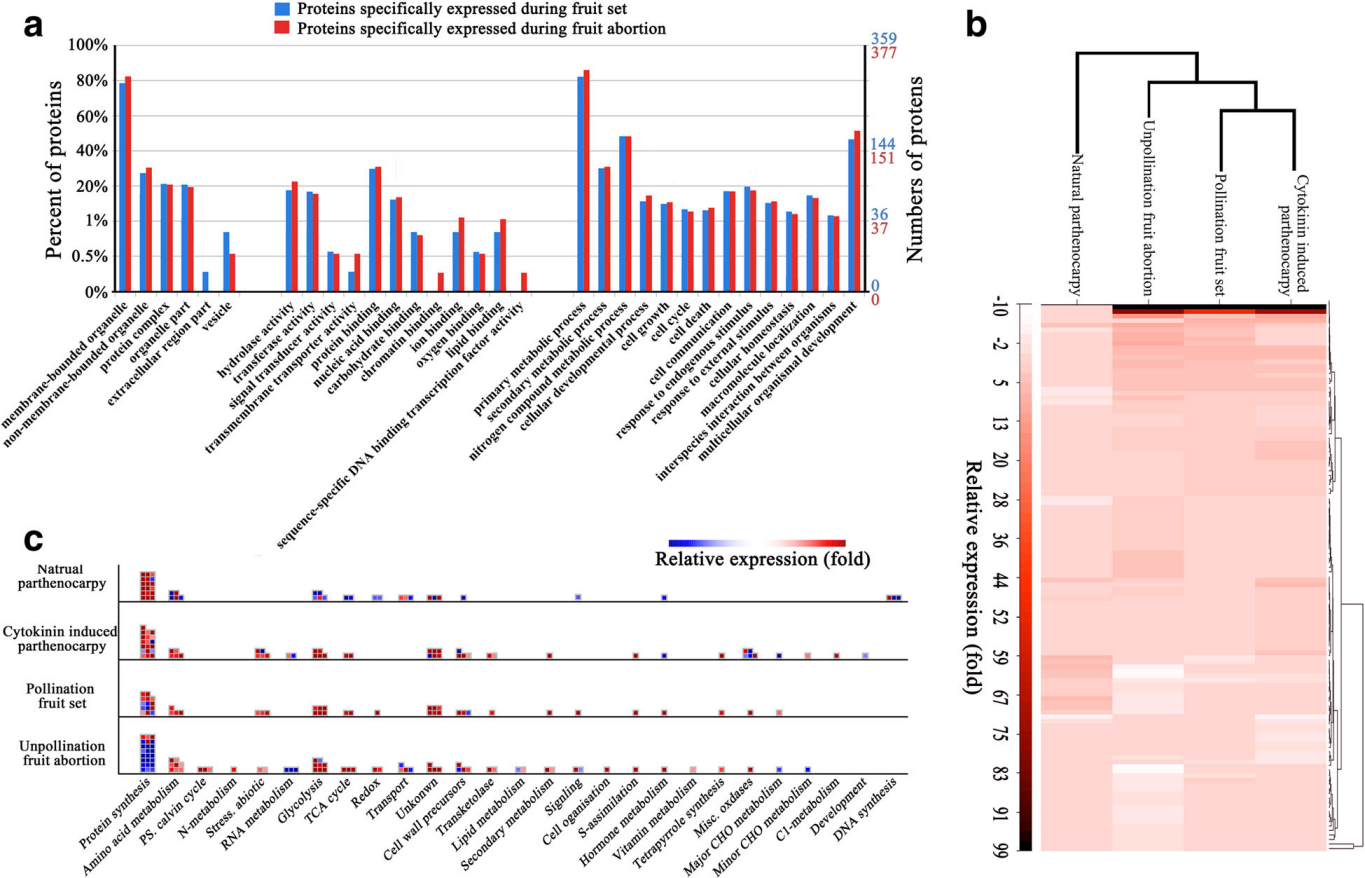
Analysis of iTRAQ-detected proteins involved in the processes of fruit set and fruit abortion. **a** A total of 359 proteins were detected from the fruits set by pollination or natural/cytokinin-induced parthenocarpy. Three hundred seventy-seven proteins were detected from abortive fruits. GO analysis suggeste that the fruit developmental proteins were involved in similar biological processe. **b** Clustering analysis of differentially expressed proteins from differently developed cucumber fruits. The cluster distance between the natural parthenocarpic and cytokinin-induced parthenocarpic fruit was farther than that between cytokinin-induced parthenocarpic and abortive fruit; KEGG analysis of DEPs from different cucumber fruits. **c** KEGG pathway analysis was performed using MapMan software (Version 3.5.1R2) according to the biological pathway maps of *Arabidopsis*

Protein homolog identification was conducted by BLASTP against the cucumber Refseq database and the *Arabidopsis thaliana* Refseq database (*E*-value <1E-10). After redundancies were removed, 683 unique proteins were identified, including 359 fruit set-related proteins (natural/cytokinin-induced parthenocarpic fruit and pollinated fruit) and 377 fruit abortion-related proteins. Gene Ontology (GO) analysis showed that the proteins detected in both set and aborted fruits were involved in similar biological processes (Fig. 2a). Proteins related to cell growth (GO:0016049), cell cycle (GO:0007049), cell death (GO:0008219) and cell communication (GO:0007154) were detected that actively expressed during fruit development, that was consistent with our previous transcriptomic study [53], suggesting the cell growth, cell cycle, cell death and cell communication related genes were actively involved in fruit development at both mRNA and protein levels.

### Identification and comparison of differentially expressed proteins involved in different fruit developmental processes

Large numbers of polymorphic SNPs (Single Nucleotide Polymorphisms), InDels (Insertion-Deletion Polymorphisms) and SVs (Structural Variations) were detected between ‘EC1’ and ‘8419 s-1’ by genome resequencing study [25]. Besides, 84 differentially expressed proteins were also identified between 0dpa fruits of ‘EC1’ and ‘8419 s-1’ (Additional file 4: Figure S3). These findings demonstrated that the genetic background of the two varieties was significantly different with each other. In order to identifiy fruit developmental DEPs (Differentially Expressed Proteins), a proteome comparison strategy was employed (Additional file 3: Figure S2). Proteomic comparisons were not conducted between cultivars, therefore the differentially expression of identified DEPs were not caused by genetic variations but only correlated with development of fruit. The false discovery rate (FDR) method (FDR ≤ 0.05, |fold ≥ 1.5|, *P*-value < 0.05) was used to determine the significance of the differential protein expression. Consequently 138 DEPs were identified and four groups of DEPs were screened (Additional file 4: Figure S3). In pollination and CP fruits, most of the DEPs were up-regulated; in contrast, more down-regulated proteins were detected in the abortive fruit of ‘8419 s-1’ and the natural parthenocarpic (NP) fruit. Clustering analysis indicated that the DEPs in CP and pollinated fruit showed similar protein expression patterns, but the protein expression profiles of the CP and NP fruits were clustered into two groups, and the cluster distance between CP and NP fruits was greater than that between CP and the abortive fruit (Fig. 2b).

Kyoto Encyclopedia of Genes and Genomes (KEGG) pathway analysis of the DEPs was conducted using MapMan software [69], according to the biological pathway maps of *Arabidopsis* (http://mapman.gabipd.org/web/guest/home). Large numbers of DEPs were shown to be involved in the biological process of protein synthesis and were mainly up-regulated during fruit set and down-regulated during fruit abortion (Fig. 2c). Transcriptome analysis has shown that amino acid metabolism, glycolysis and TCA cycle-related genes were actively expressed during fruit set [53], corroborating the present proteomic analysis, which also showed that the proteins related to these biological processes were actively expressed (Fig. 2c).

**Fig. 3:**
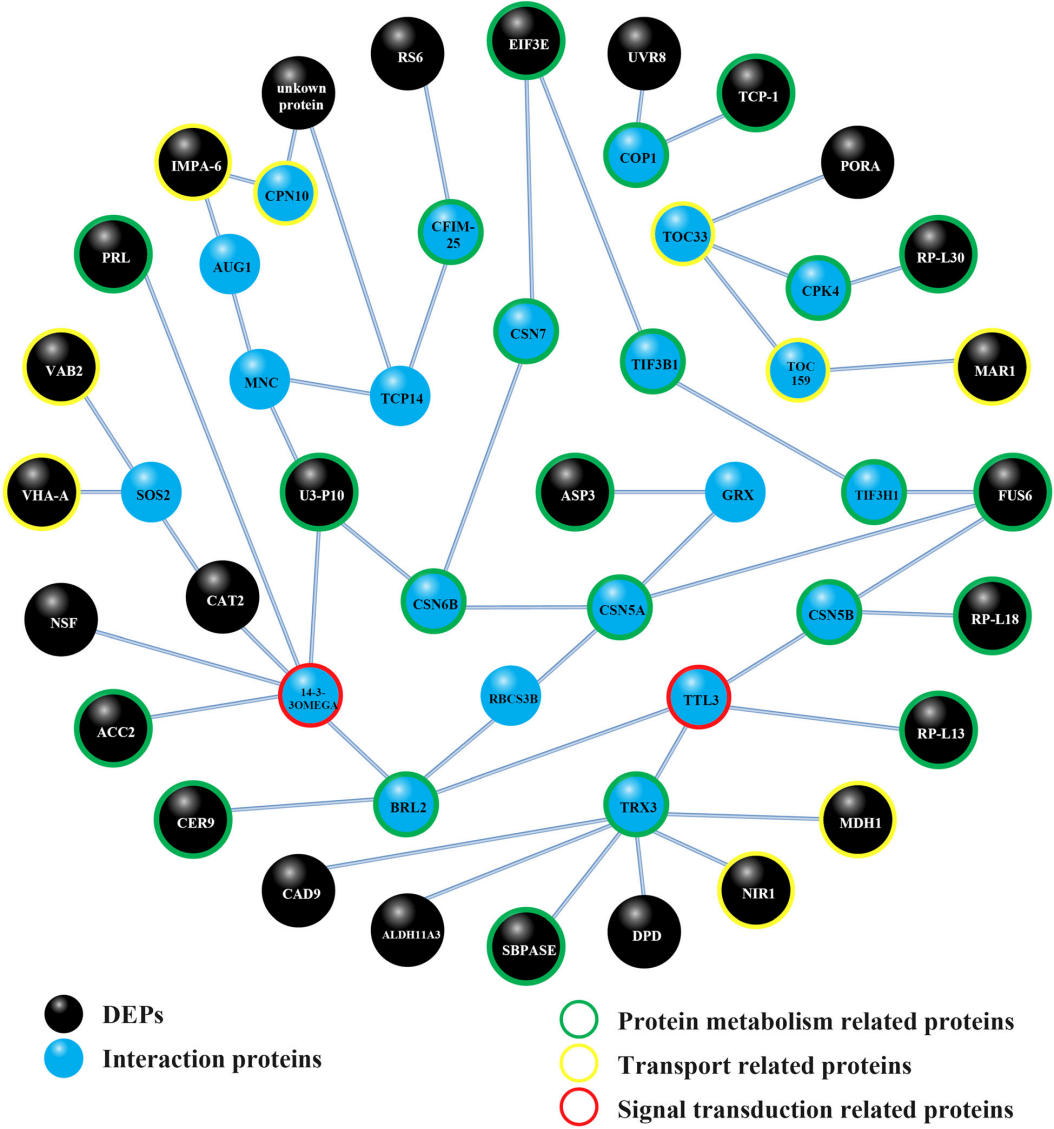
Prediction of protein-protein interactions (PPIs) in cucumber during fruit development. The PPI network was predicted by the reference proteome-wide binary PPI map of *Arabidopsis thaliana*. The PPI network consists of 30 DEPs (in black color) and 19 interaction proteins (in blue color). Within the PPI network, 23 proteins (marked by green circles) were protein metabolism (GO:0019538)-related proteins, nine transport (GO:0006810)-related proteins (marked by yellow circles) and two signal transduction (GO:0007165)-related proteins (marked by red circles). Interactions occurring in different fruit developmental processes are presented in Additional file 5: Figure S4. The annotations expressional information of the proteins are presented in Additional file 2: Table S1

The interactions between the DEPs were analyzed based on the reference proteome-wide binary protein-protein interaction (PPI) map of *Arabidopsis* [70]. A core fruit developmental PPI network was revealed, which consisted of 30 DEPs and 19 ‘bridging’ interaction proteins (Fig. 3, Additional file 2: Table S1). The proteins within the PPI network were mainly involved in the processes of protein metabolism (GO:0019538), transport (GO:0006810) and signal transduction (GO:0007165). Interactions within the PPI network occurred more frequently in fruit abortion, while the smallest scale of interactions was involved in natural parthenocarpy (Additional file 5: Figure S4). Two interaction proteins exhibited specialized expression in natural parthenocarpy: CER9 (ECERIFERUM 9, Csa7M073540.1), which is involved in cuticle metabolism and the maintenance of plant water status [71], and PRL (PROLIFERA, Csa7M407650.1), which is specifically expressed in populations of dividing cells in the sporophytic tissues of the plant body [72]. TOC159 (Csa1M229500.1, a chloroplast biogenesis-related protein), IMPA-6 (Csa1M597740.1, a nuclear import protein) and RS6 (Csa1M229500.1, a putative ovule development regulator) showed specialized up-regulation in CPPU-induced parthenocarpic fruit set (Additional file 2: Table S1).

**Fig. 4:**
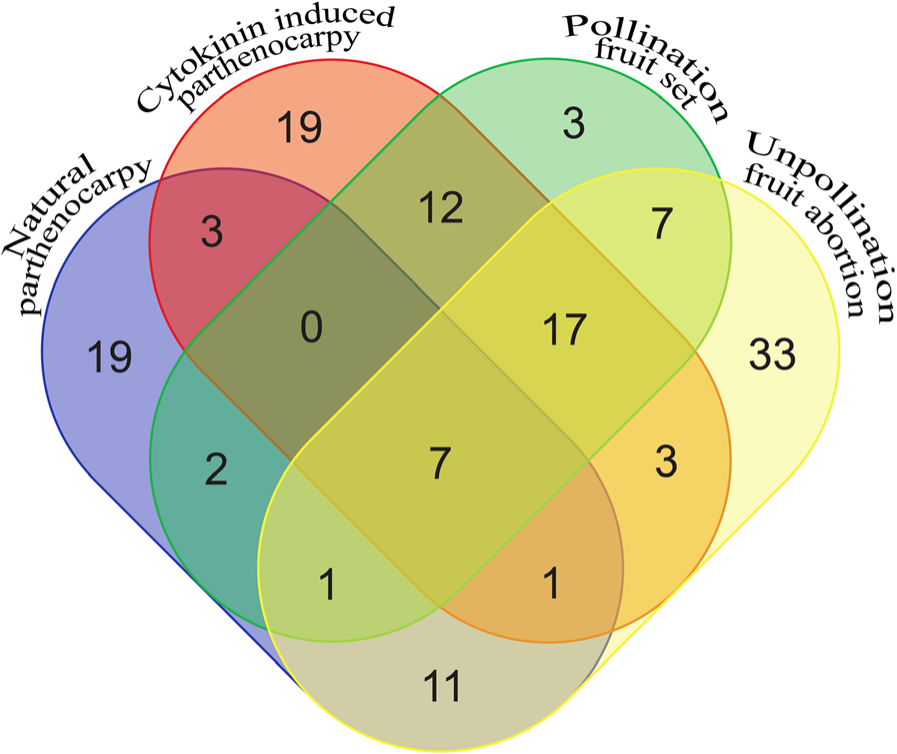
Venn diagram and relative expression of DEPs in natural and cytokinin-induced parthenocarpic, pollinated and abortive cucumber fruits. The differently expressed proteins (DEPs) from the natural parthenocarpic fruit of ‘EC1’, cytokinin-induced parthenocarpic fruit of ‘8419 s-1’, pollinated and unpollinated fruits of ‘8419 s-1’ were compared. Twelve DEPs were commonly expressed in cytokinin-induced parthenocarpic and pollinated fruits (the common DEPs are annotated in Additional file 2: Table S2). Eleven DEPs were commonly expressed in natural parthenocarpic and abortive fruits and showed opposite expression trends (Additional file 2: Table S2). Three DEPs were parthenocarpy-specialized proteins that are commonly expressed in both natural and cytokinin-induced parthenocarpic fruits (Additional file 2: Table S3). The natural and cytokinin-induced parthenocarpy-specialized proteins are individually annotated in Table 2

The common and specific DEPs in the differently developed cucumber fruits are shown in the Venn diagram in Fig. 4. No DEPs were commonly expressed in NP, CP and pollination fruit. Twelve common proteins were identified in the CP and pollinated fruits and showed similar expression patterns (Additional file 2: Table S2). Most of these CP and pollination-specialized DEPs are closely related to pollen and seed development. Eleven DEPs were commonly expressed in NP and abortive fruit, most of which were protein metabolism-related proteins and mainly involved in the biological processes of pollen germination, gametophyte and endosperm development as well as root morphogenesis; however, these proteins showed opposite expression trends during parthenocarpy and fruit abortion (Additional file 2: Table S2).

Forty-one parthenocarpy-specialized DEPs were identified, but only 3 DEPs are commonly found in the NP and CP fruits (Additional file 2: Table S3). The remaining 39 parthenocarpy-specialized DEPs were divided into two groups, of which 19 were uniquely expressed in NP fruits, while the other 19 existed only in CP fruits (Table 2). These DEPs were mainly involved in the biological processes of pollen germination, seed and seedling development, cell proliferation and programmed cell death (Table 2). The NP-specialized DEPs Csa7M073540.1 and Csa7M450640.1, which were related to cell cycle and proliferation, and Csa1M025890.1 and Csa4M036590.1, the amino acid biosynthesis-related proteins, showed dramatically up-regulated expression during fruit set (expression fold >5) (Table 2). Moreover, Csa2M139820.1, which has the putative function of translational elongation, was the only DEP that was dramatically increased in the CP fruit (Table 2).

**Table 2:** DEPs specifically expressed in natural or Cytokinin induced parthenocarpic fruit

Protein ID	Top Hit^a^	Description^b^	Relative Biological Processes^c^	Expression fold of DEPs in parthenocarpic fruit^d^
Natural parthenocarpy specialized DEPs				
Csa1M024830.1	AT1G48630.1	RACK1B, RECEPTOR FOR ACTIVATED C KINASE 1B	Seed Germination and Early Seedling Development; shoot development (GO:0048367)	−1.97
Csa1M025890.1★	AT5G46180.1	DELTA-OAT, ORNITHINE-DELTA-AMINOTRANSFERASE	Pollen germination and tube growth [86]; cellular amino acid biosynthetic process (GO:0008652)	85.50
Csa1M025980.1	AT3G10920.1	MSD1, MANGANESE SUPEROXIDE DISMUTASE 1	Seed Germination [87]; female gametophyte development; programmed cell death	−1.94
Csa2M223140.1	AT3G04840.1	40S ribosomal protein S3a-like protein	Pollen germination and tube growth [86]	2.58
Csa2M338890.1	AT1G26880.1	60S ribosomal protein L34	Pollen germination and tube growth [86]	2.99
Csa3M002370.1	AT4G35630.1	PHOSPHOSERINE AMINOTRANSFERASE 1, PSAT1	Serine biosynthesis; responses to cytokinin	−3.16
Csa3M827370.1	AT3G01280.1	VOLTAGE DEPENDENT ANION CHANNEL 1, VDAC1	Female gametogenesis; pollen germination and tube growth [86]	−2.53
Csa4M001980.1	AT1G02780.1	EMB2386, EMBRYO DEFECTIVE 2386	Pollen germination and tube growth [86]; embryonic development (GO:0009790)	3.02
Csa4M012460.1	AT1G60710.1	Aldo/keto reductase family protein	Seed Germination and Floral Development	−2.65
Csa4M036590.1★	AT4G39660.1	AGT2, ALANINE:GLYOXYLATE AMINOTRANSFERASE 2	Responses to brassinosteroids; cellular amino acid biosynthetic process (GO:0008652)	8.87
Csa4M179090.1	AT4G33680.1	AGD2, ABERRANT GROWTH AND DEATH 2	Responses to cytokinin; pollen germination and tube growth (Wang et al. [86]); cell growth	−2.44
Csa4M290220.1	AT1G09200.1	Histone H3	Cell cycle [88]; cell expansion and proliferation; male gametogenesis	−3.25
Csa4M664520.1	AT1G76550.1	Fructose-6-phosphate 1-phosphotransferase	seed development [89]; glycolysis (GO:0006096)	2.29
Csa6M193590.1	AT1G07660.1	Histone H4	Chromatin organization (GO:0006325)	−3.16
Csa6M450410.1	AT2G36460.1	FBA6, FRUCTOSE-BISPHOSPHATE ALDOLASE 6	Seed Germination [87]; responses to cytokinin	−2.03
Csa6M451470.1	AT3G52880.1	MDAR1, MONODEHYDROASCORBATE REDUCTASE 1	Pollen germination and tube growth [86]; Cell Wall Regeneration	−1.95
Csa7M073540.1★	AT4G34100.1	CER9, ECERIFERUM 9	Seed Germination; pollen germination and tube growth [86]; Cell cycle [88]	7.44
Csa7M407650.1	AT4G02060.1	PRL, PROLIFERA	Cell cycle and division [72]; GO:0007049)	3.66
Csa7M450640.1★	AT1G67120.1	MDN1, MIDASIN 1	Seed germination and seedling development; female gametophyte development; cell proliferation	87.90
Cytokinin induced parthenocarpy specialized DEPs				
Csa1M003540.1	AT4G20360.1	RABE1B, RAB GTPASE HOMOLOG E1B	Seed Germination and development [87, 89]	2.55
Csa1M031900.1	AT1G48410.1	AGO1, ARGONAUTE 1	Fruit development; cell division	−1.67
Csa1M042700.1	AT3G18080.1	BGLU44, B-S GLUCOSIDASE 44	Female gametophyte development; Cell wall proteins	−1.80
Csa1M229500.1	AT5G20250.1	DIN10, DARK INDUCIBLE 10	Seed germination and seedling development; pollen germination and tube growth [86]	1.78
Csa1M573730.1	AT5G56680.1	EMB2755, EMBRYO DEFECTIVE 2755	Gametogenesis and embryo development (GO:0009793)	1.95
Csa1M597740.1	AT5G20720.1	CPN21, CHAPERONIN 20	Seed development [89]; pollen germination and tube growth	1.55
Csa1M604600.1	AT1G50480.1	THFS,10-FORMYLTETRAHYDROFOLATE SYNTHETASE	Seed development [89]; responses to cytokinin	2.88
Csa2M139820.1★	AT1G07920.1	EF1α, Elongation factor 1-alpha	Pollen development; translational elongation (GO:0006414)	6.08
Csa2M264020.1	AT4G34880.1	GAtA, Glutamyl-tRNA (Gln) amidotransferase subunit A	Translation (GO:0006412)	2.70
Csa2M350200.1	AT1G24510.1	TCP-1/cpn60 chaperonin family protein	Plant cell death [90]	1.95
Csa4M094000.1	AT3G29360.1	UGD2, UDP-GLUCOSE DEHYDROGENASE 2	Pollen germination and tube growth [86]; cell wall organization (GO:0007047)	3.98
Csa4M496230.1	AT5G63860.1	UVR8, UVB-RESISTANCE 8	Cell cycle (GO:0007049)	−3.02
Csa5M623870.1	AT5G07030.1	Aspartic proteinase nepenthesin-1	Re-arrangements of cell wall [91]	−2.44
Csa5M644550.1	AT3G02530.1	TCP-1/cpn60 chaperonin family protein	Plant cell death [90]	1.85
Csa6M439410.1	AT5G19440.1	Cinnamoyl CoA reductase-like protein	Seed development [89]; lignin biosynthetic pathway	−4.09
Csa7M048110.1	AT3G14940.1	PPC3, PHOSPHOENOLPYRUVATE CARBOXYLASE 3,	Development of male gametophyte; Cell cycle [88]	2.75
Csa7M075590.2	AT5G42650.1	AOS, ALLENE OXIDE SYNTHASE	Floral organ development; defense response (GO:0006952)	−2.65
Csa7M390010.1	AT3G02080.1	40S ribosomal protein S19	Translation (GO:0006412)	2.55
Csa7M405310.1	AT4G02290.1	GH9B13, GLYCOSYL HYDROLASE 9B13	Root development; cell wall organization (GO:0007047)	−2.42

^a^Homologous search was conducted by BLASTP against the *Arabidopsis* Refseq database (http://www.arabidopsis.org/index.jsp). *E*-value was set to <1E-10. The *Arabidopsis* gene ID with highest score is picked for further analysis

^b^The proteins were annotated based on the public databases: *Arabidopsis* database (http://www.arabidopsis.org/index.jsp) and cucumber Refseq database (http://cucumber.genomics.org.cn/page/cucumber/index.jsp)

^c^The proposed biological processes were refer from the GO terms and related research reports of the top hit *Arabidopsis* genes

^d^The expression fold was calculated as the ratio of the protein expression in 2 dpa fruits vs. protein expression in 0 dpa fruit, *P*-value <0.05; The dramatically increased (fold >5) DEPs were marked with stars

### Expression analysis of NP- and CP-specialized proteins in response to phytohormones

**Fig. 5:**
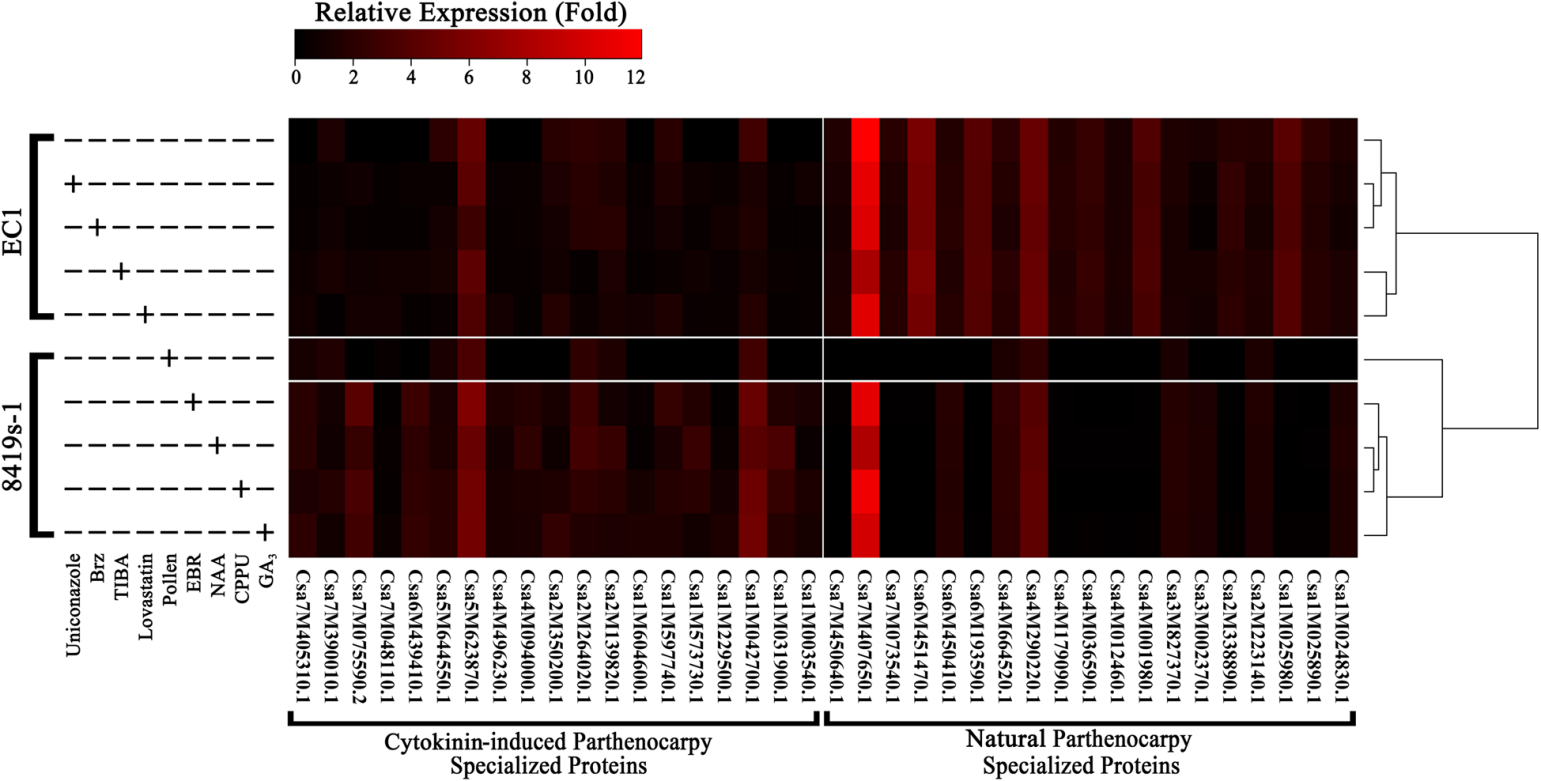
Transcription analyses of parthenocarpy-specialized DEPs in different types of parthenocarpic fruits. Ovaries of ‘EC1’ and ‘8419 s-1’ were treated by hormones and hormone inhibitors separately as described in the materials and methods section. Thus, different types of parthenocarpic fruits were induced (Table 1, Additional file 1: Figure S1). Transcription analysis was conducted by quantitative real-time PCR. Transcriptional profiles of the two groups of parthenocarpy-specialized DEPs in different types of parthenocarpic fruits were clustered. The experiment was repeated three times. Each value represents the mean ± SE of three replicates

The transcription profiles of the parthenocarpy-specialized DEPs in the hormone- and hormone inhibitor-treated fruits were compared (the treated fruits are described in the 1st part of the results section). Consistent with the result of iTRAQ, the specialized DEPs from both parthenocarpy groups showed inactive transcription during pollinated fruit set (Fig. 5, middle panel). The CP-specialized DEPs were actively expressed in the cytokinin-induced parthenocarpic fruits of ‘8419 s-1.’ However, in the parthenocarpic fruits of ‘EC1,’ including the hormone inhibitor-treated but not blocked parthenocarpic fruits, most proteins showed decreased expression (Fig. 5, left column of the panel). In contrast, the NP-specialized DEPs showed up-regulated expression in parthenocarpic fruits of ‘EC1’, whereas many of these DEPs were silenced in the cytokinin-induced parthenocarpic fruits (Fig. 5, right column of the panel). Although a few of the NP-specialized DEPs were differentially transcribed in cytokinin-induced parthenocarpic fruits, the expression levels of the genes in these fruits were the same as those in the parthenocarpic fruits of ‘EC1.’ Moreover, similar transcription patterns of NP-specialized DEPs between the natural parthenocarpic and unblocked natural parthenocarpic fruits were found, indicating that the transcription of these proteins could not be affected by hormone inhibitors (Fig. 5, top right of the panel). These findings, to some extent, indicated that the transcription of NP-specialized DEPs was not sensitive to hormones or hormone inhibitors.

**Fig. 6:**
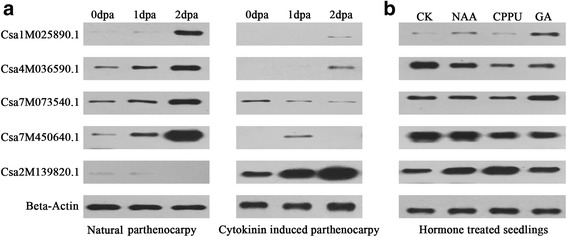
Western blot analysis of the parthenocarpy-specialized proteins that were actively expressed during NP and CP fruit set. iTRAQ result showed that Csa1M025890.1, Csa4M036590.1, Csa7M073540.1, and Csa7M450640.1 were dramatically increased in natural parthenocarpic fruits, while Csa2M139820.1 was highly increased in cytokinin-induced parthenocarpic fruits (Table 2; marked by solid stars, >5-fold). The expression patterns of these proteins were further analyzed by western blotting. **a** Expression analysis of the parthenocarpy-specialized proteins during NP and CP fruit set individually. **b** Expression analysis of the parthenocarpy-specialized proteins in response to hormone treatments in seedlings. The cucumber beta-actin (Csa5M182010.1) was used as reference protein for Western blotting. The experiment was repeated three times. The band intensity analysis of western blots was conducted using ImageJ (Version 1.4), and the data are presented in Additional file 6: Figure S5A. **CK**: Seedlings without phytohormone treatment; **NAA**: treated with 50 μM NAA; **CPPU**: treated with 10 μM CPPU; **GA**: treated with 10 μM GA_3_

iTRAQ showed that four NP-specialized proteins—Csa7M073540.1, Csa7M450640.1, Csa1M025890.1 and Csa4M036590.1—showed a high abundance in expression during parthenocarpy (expression >5-fold; Table 2, marked by stars). To further investigate the expression characteristics of the active parthenocarpy-specialized proteins, we conducted a western blot analysis. Consistent with the results of iTRAQ, the NP-specialized proteins were up-regulated during natural parthenocarpic fruit set but inactively expressed in the cytokinin-induced fruit, while the NP-specialized protein was not detected in the NP fruit but was actively expressed in the CP fruit (Fig. 6a). Auxins, cytokinins and gibberellins are essential fruit developmental phytohormones, which can induce parthenocarpy in cucumber. The expression patterns of the parthenocarpy-specialized proteins responding to these hormones were also investigated (Fig. 6b). Our results showed that the treatment with endogenous hormones NAA, CPPU and GA3 increased the expression of the CP-specialized protein Csa2M139820.1 but decreased the expression of Csa4M036590.1 and Csa7M450640.1 (NP-specialized proteins). Although the NP-specialized proteins Csa1M025890.1 and Csa7M073540.1 were up-regulated by GA3, they were not significantly changed by NAA and CPPU (Fig. 6b, Additional file 6: Figure S5A).

**Fig. 7:**
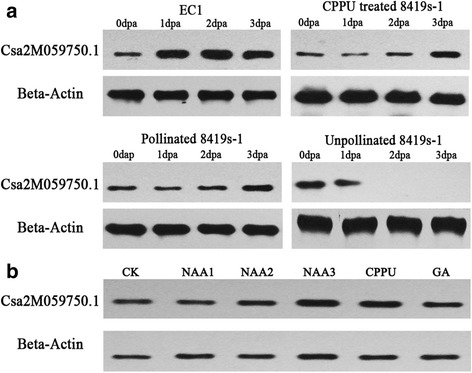
Expression analysis of Csa2M059750.1 during different fruit developmental processes and the response to phytohormone treatments. Csa2M059750.1 was considered a candidate parthenocarpy regulatory protein by combined analysis of iTRAQ and genetic mapping results (Wu et al. [25]). The protein expression of Csa2M059750.1 was analyzed by western blotting. **a** The expression of Csa2M059750.1 during fruit development; **b** The expression of Csa2M059750.1 after phytohormone treatment in cucumber seedlings. The cucumber beta-actin (Csa5M182010.1) was used as a reference protein for western blotting. The experiment was repeated three times. The band intensity analysis of western blots was recorded using ImageJ (Version 1.4), for which the data are presented in Additional file 6: Figure S5B. **CK**: Seedlings without phytohormone treatment; **NAA1**: treated with 5 μM NAA; **NAA2**: treated with 10 μM NAA; **NAA3**: treated with 50 μM NAA; **CPPU**: treated with 10 μM CPPU; **GA**: treated with 10 μM GA_3_

The protein expression pattern of Csa2M059750.1 was also analyzed, which was up-regulated during natural parthenocarpy and down-regulated in abortive fruit (Additional file 2: Table S3). Csa2M059750.1 was the unique DEP located in the chromosome region of the major parthenocarpic QTL *Parth2.1* of ‘EC1’ [25]. Western blot analysis showed that Csa2M059750.1 was degraded during fruit abortion but actively expressed during fruit set. However, in contrast to the natural parthenocarpic fruit set, the increasing expression of Csa2M059750.1 was delayed in the pollinated and CP fruits until 3 dpa (Fig. 7a). Western blot analysis also showed that the expression of Csa2M059750.1 was not significantly affected by hormones, indicating that Csa2M059750.1 may be a hormone-insensitive protein (Fig. 7b, Additional file 6: Figure S5B).

## Discussion

Although studies on parthenocarpy have been conducted for over 100 years, current understanding of parthenocarpy remains at a nascent stage. Cucumber is emerging as a model for the *Cucurbitaceae* family. The rich parthenocarpic germplasm of cucumber offers an opportunity to investigate the coordination and communication of environmental factors, hormone signals and genes during parthenocarpy. In this study, we investigated the proteomes of cucumber fruits to help improve the global understanding of parthenocarpy.

### Post-translational regulation of fruit development in cucumber

Proteins are executors with a vast array of functions within organisms. The translational and post-translational regulations of proteins such as protein synthesis, proteolysis, glycosylation, phosphorylation and folding are essential for plant development. Studies on hormone-dependent biological processes have revealed that post-translational regulations frequently occur during fruit development. For instance, in the absence of auxins, the function of ARFs (auxin response factors) was inhibited via heterologous dimerization with Aux/IAA. However, in the presence of auxin, the ARFs dissociated from Aux/IAA proteins, whose targeting and degradation were mediated by the E3 ubiquitin ligase SCF^TIR1/AFB^, and consequently stimulated fruit set [5, 73–77]. The fruit developmental responses to ethylene were mediated by the SCF^EBF1/EBF2^-dependent proteolysis of EIN3 (Ethylene insensitive 3) [78]. Furthermore, the function of EIN3 was regulated by the MAPK-dependent phosphorylation within the EPR1 domain of the transcriptional factor [79]. In addition, our previous transcriptome study confirmed that glycosylation reactions were dramatically active throughout fruit development in cucumber [53]. In the present proteomic study, the protein folding-related proteins were up-regulated in both pollinated and parthenocarpic fruits, including Csa1M255160.1, which was annotated as a TCP-1 (T-COMPLEX PROTEIN 1 ALPHA SUBUNIT), and was actively expressed in natural and cytokinin-induced parthenocarpic fruits (Additional file 2: Table S3). Moreover, Csa2M099450.1, also defined as a TCP-1-like protein, showed specialized expression in pollinated and cytokinin-induced parthenocarpic fruits (Additional file 2: Table S1).

Many lines of evidence indicate that protein synthesis/degradation may function during cell growth [80, 81]. The present proteomic study showed that over 30% of the differentially expressed proteins during the cucumber fruit development were related to the biological process of protein metabolism (Fig. 2c). Within the predicted IPP network, nearly half of the interaction proteins were protein metabolism-related proteins, such as the three TIF (TRANSLATION INITIATION FACTOR) proteins involved in the initiation phase of eukaryotic translation, four CSN (COP9 signalosome) multi-proteins that functioned in the ubiquitin–proteasome pathway, and three ribosomal proteins (Additional file 2: Table S1). Protein metabolism related proteins were commonly expressed in unpollinated and natural parthenocarpic fruit (Additional file 2: Table S2). The opposite expression patterns of the common proteins indicated the fate (set or abortion) of the mature ovary in cucumber, which was determined by protein metabolism pathways.

### The cues of hormone-independent parthenocarpy in cucumber

Growth measurement showed that natural and cytokinin-induced parthenocarpic fruits presented similar growth curves (Fig. 1a). However, clustering analysis showed that the protein expression profiles of the CP and NP fruits were quite different from each other. Moreover, the cluster distance between the CP and NP fruits was greater than that between the CP fruit and the abortive fruit (Fig. 2b). The specialized proteins expressed in the parthenocarpic fruits were divided into two individual groups, which were separately involved in the NP and CP fruit set, as shown in the Venn diagram (Fig. 4; Table 2). These findings suggested that there may be individual parthenocarpic pathways in cucumber.

Gustafson [54, 55] proposed that plants produce parthenocarpic fruits because the ovary contains enough auxins to promote fruit initiation. Since then, many studies have confirmed that parthenocarpy is a phytohormone-dependent biological process. In cucumber, polar auxin transport-blocking experiments have shown that parthenocarpy could be triggered by the sufficient accumulation of auxin in the ovary [44]. Moreover, the application of exogenous hormones such as auxins, cytokinins, gibberellins and brassinosteroids could induce parthenocarpy [51]. In this study, hormone measurement showed that the endogenous hormone levels increased during fruit set but decreased during fruit abortion in ‘8419 s-1’ (Fig. 1b). However, the endogenous hormone levels were relatively low and remained stable during natural parthenocarpic fruit set in ‘EC1’ compared with ‘8419 s-1.’ Moreover, the NP fruits showed a broad resistance to hormone inhibitors (Fig. 1b; Table 1; Additional file 1: Figure S1), indicating the existence of a hormone-independent parthenocarpic mechanism in ‘EC1.’ This speculation was supported by expression analysis in the parthenocarpy-specialized proteins, whereby the NP-specialized proteins performed hormone-insensitive transcriptional and translational functions (Figs. 5, 6 and 7; Additional file 6: Figure S5).

### Inhibiting the regulation of fruit abortion in cucumber

Dormant fruits, as a result of first-fruit inhibition or nutritional stress, can always be observed in the field [25, 26]. However, the dormant state of these fruits usually leads to fruit abortion in a short time (2 days at most). Although natural parthenocarpic fruits of ‘EC1’ could not be blocked by hormone inhibitors, these treated fruits stayed in a dormant state for a long time (more than 4 days) (Additional file 1: Figure S1). We speculated that inhibitory regulations of fruit abortion might exist in ‘EC1’, causing the fruits to maintain a dormant state. Coincidentally, the common proteins that detected in the NP fruits of ‘EC1’ and the abortive fruit of ‘8419 s-1,’ showed opposite expression trends. Most of these proteins were down-regulated during fruit abortion but up-regulated during NP fruit set (Additional file 2: Table S2). Besides, ethephon treating experiments showed that although fruit abortion of the non-parthenocarpic cultivars was accelerated by ethephon which had no effect on fruit development of ‘EC1’, further suggesting inhibitory regulations of fruit abortion might exist in ‘EC1’. Conversely, hormone inhibitor-induced dormant state in ‘EC1’ indicated that hormone stimuli might be required for fruit expansion in either parthenocarpic cultivars or non-parthenocarpic cultivars.

## Conclusions

**Fig. 8:**
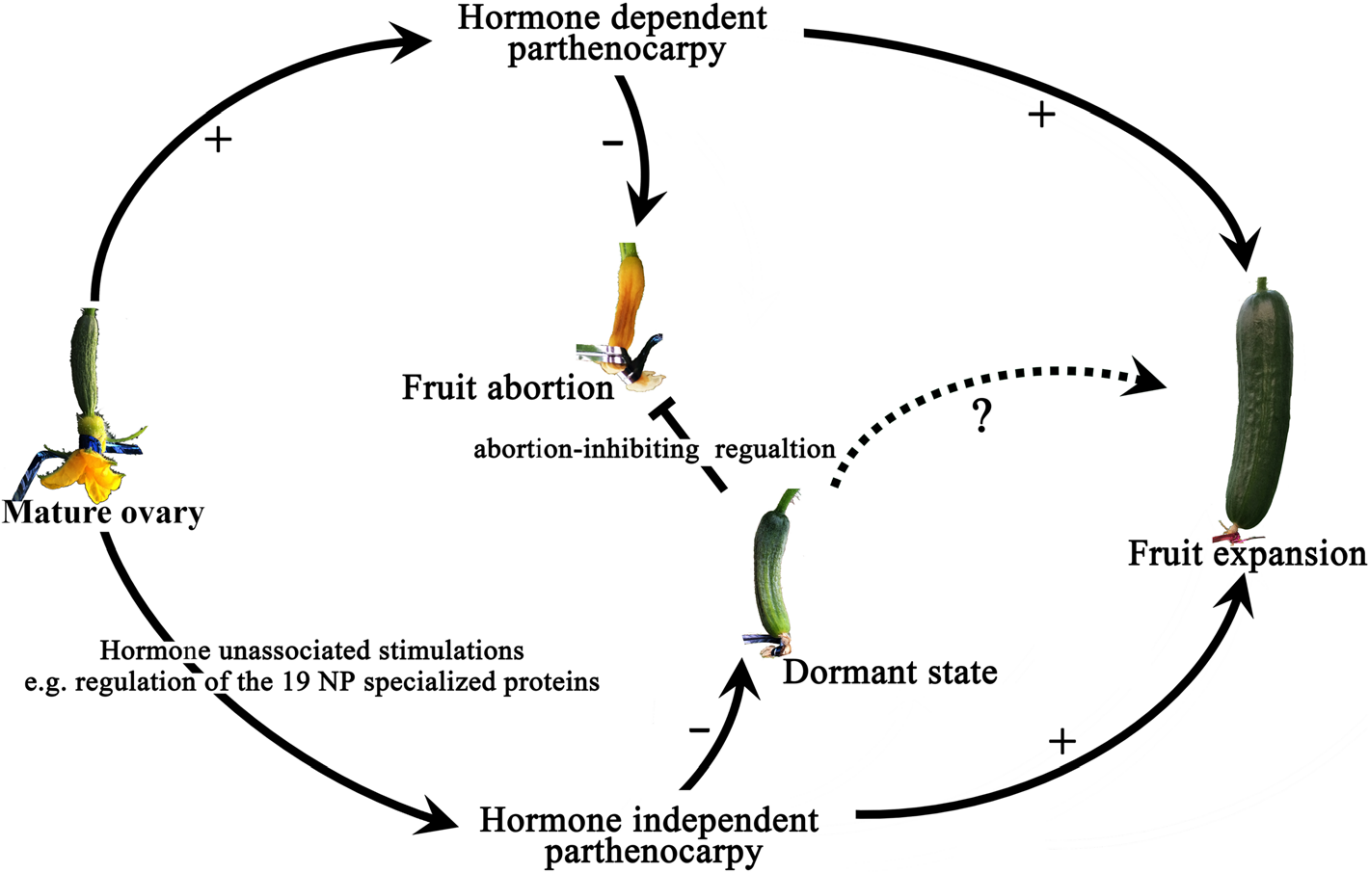
A proposed model for parthenocarpy in cucumber. Proposed model illustrating the working hypothesis of parthenocarpy, which can be promoted by either hormone-dependent or -independent pathways. The hormone-unassociated stimulations may be regulated by the NP-specialized proteins (Table 2) because of their hormone-insensitive expression characteristics. In the presence of sufficient hormone levels (endogenous or exogenously supplied), the parthenocarpic young fruits can continue to grow. However, in the absence of hormones, hormone-dependent parthenocarpic fruits will return to the fruit abortion pathway, while the hormone-independent parthenocarpic fruits will stay in a dormant growth state that may be caused by abortion-inhibiting proteins. Whether the dormant fruits can restart growth or be artificially induced remains unclear. **‘+’**: in the presence of hormones; **‘-’**: in the absence of hormones. The plant images were taken by JL in a greenhouse of Jiangpu experimental station of Nanjing Agricultural University

Based on the evidence provided in this study, a working hypothesis for the cucumber parthenocarpic fruit set was proposed (Fig. 8), whereby parthenocarpy in cucumber may be promoted by a ‘parallel switch,’ namely, hormone-dependent and hormone-independent pathways. During hormone-independent parthenocarpy, fruit set was promoted by hormone-insensitive regulatory proteins, such as the NP-specialized proteins in ‘EC1.’ In the presence of sufficient hormones, young fruits formed through both hormone-dependent and -independent pathways could continuously grow to maturity. In the absence of hormones, the development of hormone-sensitive fruits proceeds to fruit abortion, whereas the hormone-insensitive fruits remain in a dormant state because of the increasing expression of abortion-inhibiting proteins. However, the expansion of dormant fruits and their further promotion are unknown. Although the accurate regulation of parthenocarpy in cucumber remains unclear, our studies provide a theoretical framework for understanding the mechanism of parthenocarpy for its application in agricultural production.

## Methods

### Plant material and growth conditions

In this study, the cucumber cultivar ‘EC1’ was used as a parthenocarpic sample (Gynoecious inbred line, European Glasshouse type, parthenocarpic rate ≥ 95%) and ‘8419 s-1’ as a non-parthenocarpic sample (Monoecious inbred line, European Glasshouse type, the rare occurrence of parthenocarpy is occasionally observed in the senescence phase of the cultivar). Plants were grown in a greenhouse at Nanjing Agricultural University with a 14 h photoperiod, a mean daily air temperature of 28/20 °C (day/night).

### Phytohormone measurement

Phytohormones were separately analyzed through ELISA using IAA, ZR and GA3 ELISA Kits (Sangon Biotech Company) based on Weiler’s method [82]. The results are presented as the mean ± SE (*n* = 10) with three technical replicates.

### Ovary treatments

The female flowers (at the 12-15th node of the main stem) of the above cucumber cultivars were previously trapped with bags in order to prevent pollen contamination on the day before anthesis. When anthesis, the trapped ovaries were treated separately: keeping trapping (unpollination), hand pollination [34] and CPPU treatment. CPPU (N-(2-chloro-4-pyridyl)-N′-phenyl urea) is a kind of synthetic cytokinin which could induce parthenocarpy in cucumber. For CPPU treatment, 20 μL CPPU solution (100 mg/L) was sprayed on the surface of the ovaries. All the treated ovaries were harvested at 0, 1, 2 and 3 dpa (days-post-anthesis). Thirty ovaries of each treatment were ground into powder with liquid nitrogen and mix as a sample pool for iTRAQ and western blot analysis.

The trapped ovaries of ‘EC1’ and ‘8419 s-1’ were also treated with phytohormones (NAA, 1-naphthaleneacetic acid, 50 mg/L; CPPU, 100 mg/L; GA3, Gibberellin A3, 50 mg/L; EBR, Epi-Brassinosteroids, 10 mg/L; Ethephon, 100 mg/L) and hormone inhibitors (TIBA, 2,3,5-triodobenzoic acid, inhibitor of auxin, 50 mg/L; Lovastatin, inhibitor of cytokinin, 50 mg/L; uniconazole, inhibitor of gibberellin 50 mg/L; Brz, Brassinazole, inhibitor of Brassinosteroids, 10 mg/L; STS, silver thiosulphate, inhibitor of ethylene, 0.25 mM). The exogenous phytohormones and hormone inhibitors were separately sprayed on the surface of the ovaries at 0dpa. Active pollens and irradiated pollens (γ-ray irradiation at a dose of 200Gy) also used to treat the 0dpa ovaries by hand-pollination. After spaying and hand-pollination, the ovaries were trapped again. The weight, length and diameter of the ovaries were measured at 4dpa. The experiments were repeated three times (*n* = 30). The treated ovaries were also harvested at 2dpa, of which the RNA was isolated for qRT-PCR analysis.

For western blotting, the seedlings of ‘8419 s-1’ (at the three true leaf stage) were also treated with exogenous phytohormones (50 μM, 10 μM, or 5 μM NAA; 10 μM CPPU and 10 μM GA3) by spraying the solutions on the surface of the true leaves. After growing in the growth chamber with a 14 h photoperiod and 25 °C for 24 h, in total 30 true leaves from five individual plants by same treatment were collected and mixed by grinding in liquid nitrogen, then stored at −80 °C before protein extraction.

### Protein extraction and quantization

Approximately 1 g of powdered sample was mix with 3 mL extraction buffer [500 mM Tris-HCl (pH 7.5), 150 mM NaCl, 50 mM ethylene diaminetetraacetic acid (EDTA), 1% Triton-X-100, 2 mM dithiothreitol (DTT), 2 mM phenylmethanesulfonyl fluoride (PMSF)]. Protein extraction was performed using the methods described by Omar et al. [83]. The protein precipitation was collected and washed with cold methanol containing 10 mM DTT three times, cold acetone containing 10 mM DTT twice and then dried by vacuum freeze. The extracted proteins were quantified by using the Bradford method [84].

### Protein digestion and iTRAQ labeling

One hundred micrograms Proteins from each samples were precipitated with five volume of cold acetone at −20 °C for 1, centrifuged by 12,000 rpm for 15 min at 4 °C, and dried by vacuum freeze dryer (Thermo savant, USA). Pellets were dissolved in the dissolution buffer with reducing reagent described in iTRAQ Reagent 8-Plex kit (Applied Biosystems, USA), and alkylated by cysteine-blocking reagent according to the manufacturer’s instructions [85]. After digestion with 50 μl of 50 ng/μl sequence grade modified trypsin (Promega, USA) solution overnight at 37 °C, the peptide samples were labeled. The samples were labeled with the iTRAQ tags as described in Additional file 1: Figure S1.

### SCX chromatography and LC–MS/MS analysis

The vacuum dried iTRAQ labeled samples were re-suspended with 100 μl SCX (Strong cation exchange) buffer A (10 mM ammonium formate, 20% ACN (acetonitrile), pH 2.8) and fractionated using a Poly-SEA HPLC (High Performance Liquid Chromatography) column (2.0 × 150 mm, 5 μm particle size, 300 Ǻ pore size) using at a flow rate of 0.3 ml/min on the Agilent 1200 HPLC System (Agilent, USA). The 50 min HPLC gradient consisted of 100% buffer A (10 mM ammonium formate, 20%ACN, pH 2.8) for 5 min, 0–50% buffer B (500 mM ammonium formate, 20%ACN, pH 2.8) for 25 min, then 50–80% buffer B for 15 min, followed by 80–100% buffer B for 10 min, and lastly 100% buffer B for 15 min. Chromatograms were recorded at 215 and 280 nm. All the collected fractions were vacuum dried, and re-suspended with Nano- RPLC (Reversed Phase Liquid Chromatography) buffer A (0.1% FA, folic acid; 2%ACN). Samples were desalted with C18 nanoLC trap column (100 μm ID × 3 cm, 3 μm particle size, 150 Ǻ pore size) and Nano-RPLC buffer A (0.1%FA, 2%ACN) at 2 μl/min for 10 min for LC–MS/MS analysis.

The mass spectroscopy analysis was performed using a Triple TOF 5600 System (AB SCIEX, USA), coupled with the Eksigent nanoLC-Ultra™ 2D System (AB SCIEX, USA).The iTRAQ labeled peptides were separated using an analytical ChromXP C18 column (75 μm ID × 15 cm, 3 μm particle size, 120 Å pore size) (New Objectives, USA) with a nanospray emitter (2500 V, 30 PSI (pounds per square inch) curtain gas, 5 PSI nebulizer gas, 150 °C interface heater temperature) (New Objectives, USA), and analyzed by LC-MS/MS. A rolling collision energy setting was applied to all precursor ions for collision-induced dissociation (CID). For information dependent acquisition (IDA), survey scans were acquired in 250 ms and as many as 35 product ion scans were collected if they exceeded a threshold of 150 counts per second (counts/s) with a 2+ to 5+ charge-state. The total cycle time was fixed to 2.5 s. Dynamic exclusion was set for one-half of peak width (18 s), and then the precursor was refreshed off the exclusion list. The peak areas of the iTRAQ reporter ions reflect the abundance of the proteins in the samples.

### Protein identification

Mass spectrometric data was processed with Protein Pilot Software v. 4.0 (AB SCIEX, USA) against *Cucumber* database using the Paragon algorithm, and further processed by a Pro Group algorithm where isoform-specific quantification was adopted to trace the differences between expressions of various isoforms which was applied to the peptide identification. Protein identification was performed with emphasis on biological modifications option. Database search parameters were the followings: instrument was TripleTOF 5600, iTRAQ 8-plex quantification, cysteine modified with iodoacetamide, biological modifications were selected as the ID focus, trypsin digestion. An automatic decoy database search strategy was employed to estimate the false discovery rate (FDR) using the Proteomics System Performance Evaluation Pipeline Software (PSPEP) t was integrated in the Protein Pilot Software. In this study, only protein quantification data with the value of global FDR ≤0.05 were chosen for further analysis, and proteins with a |fold change ≥1.5| were considered to be significantly differentially expressed.

### Quantitative real time PCR

Proteins based on their differential expression patterns revealed by iTRAQ were selected for verification by Quantitative real-time PCR (qRT-PCR) with primers designed using Primer 5.0 software (Additional file 2: Table S4). Total RNA of the samples described above was extracted by Trizol (Invitrogen, USA). After extraction, total RNA was treated with DNase I (Fermentas, UK) according to the manufacturer’s protocol. First-strand cDNA synthesis was carried-out using the PrimeScript™ RT-PCR Kit (TaKaRa, Japan). The real-time qRT-PCR was accomplished in a thermal cycler and analyzed by an IQ5 multicolor Real-time PCR detection system (Bio-Rad, USA). To determine relative fold differences for each sample in each experiment, the CT values were normalized using *Cs-actin* as an internal control and calculated relative to a calibrator using the formula 2-^△△Ct^. The experiment was repeated three times.

### Western blot analysis

Proteins extracted from ovaries and leaves as previously described were mixed with protein lysis buffer at 4 °C. Total protein lysis was boiled at 98 °C for 10 min and separated with 10% SDS-PAGE (Sodium dodecyl sulfate-polyacrylamide gel electrophoresis), then transferred to nitrocellulose (NC) membranes (GE Hybond, USA) with semi-dry approach. After 2 h of blocking with 5% milk in TBST (Tris Buffered Saline with Tween), membranes were incubated with polyclonal antibodies against the proteins of cucumber that were raised in rabbit by synthetic peptides (Lufei, P.R.China; Additional file 2: Table S5). Polyclonal antibody against cucumber beta-actin was used as internal control. The antibodies were used at 1:300 dilutions. Membranes were incubated with goat anti-rabbit IgG (Proteintech, USA) at 1:2000 dilutions in TBST for 2 h, after that membranes were washed with TBST for five times. Signals were detected by using enhanced electro-chemiluminescence (Beyotime, P.R. China).

## Additional files

Additional file 1: Figure S1. The typical phenotypes of the treated ovaries of ‘EC1’ and ‘8419 s-1’ at 4 dpa. (DOCX 196 kb)

Additional file 2: Table S1. The annotation and expressional information of the proteins within the protein-protein interactions (PPIs) which presented in Fig. 5. **Table S2.** DEPs commonly expressed in pollinated and Cytokinin induced parthenocarpic fruit (I), and DEPs commonly existed in unpollinated and natural parthenocarpic fruit (II). **Table S3.** DEPs commonly expressed in natural and cytokinin induced parthenocarpic fruits. **Table S4.** The primers of natural and cytokinin induced parthenocarpy specialized protein encoding genes for qRT-PCR. **Table S5.** The antigenic peptides of parthenocarpy specialized proteins for producing rabbit polyclonal antibodies. **Table S6.** The measurement of ovary weight, length and diameter after treated by ethephon and ethylene inhibitors.The treatments were conducted at the anthesis day (0 dpa), and the measurements were conducted at 4 dpa. Means (±SE) of three independent experiments were calculated.Letters indicate differences between the treated ovaries with statistical significance at *P* ≤ 0.05 (t-test).Detail information of EC1 and 8419 s-1 was described in M&M section. Both cucumber cultivars CC3 and CCMC were non-parthenocarpic, monoecious inbred lines, Asia ecotype. (XLSX 35 kb)

Additional file 3: Figure S2. The analytical strategy of the iTRAQ based proteome analysis of cucumber fruits. Total proteins of each sample were extracted and labeled separately with tags (113 to 118). Proteomic analysis was conducted by iTRAQ. The differentially expressed proteins (DEPs) were identified by comparing proteomes of 0 and 2 dpa ovaries of each treatment. **CK1 and CK2:** Control sample 1 and Control sample 2; **NP:** natural parthenocarpic fruits of EC1; **Unp:** Unpollination fruits of 8419 s-1 (fruit abortion); **P:** pollination fruits of 8419 s-1; **CP:** Cytokinin induced parthenocarpic fruits of 8419 s-1. Bar = 20 mm. (DOCX 357 kb)

Additional file 4: Figure S3. Statistics of differentially expressed proteins during different fruit developmental processes of cucumber. **NP:** natural parthenocarpic fruits of EC1; **Unp:** Unpollination fruits of 8419 s-1 (fruit abortion); **P:** pollination fruits of 8419 s-1; **CP:** Cytokinin induced parthenocarpic fruits of 8419 s-1. (DOCX 247 kb)

Additional file 5: Figure S4. Protein interactions occurred in different fruit developmental processes based on the predicted PPIs network. The interactions marked in red color means the interactions involved in Cytokinin induced parthenocarpy (A), natural parthenocarpy (B), pollination fruit set (C) and unpollination fruit abortion (D). (DOCX 349 kb)

Additional file 6: Figure S5. Band intensity analysis of western blotting by using the software ImageJ. The relative expression fold of each parthenocarpy specialized protein was calculated by the formula: (band intensity after hormone treatments/band intensity without hormone treatment)/(band intensity of beta-actin in hormone treated sample/band intensity of beta-actin in untreated sample). Each value represents the mean ± SE of three Western blotting replicates. (DOCX 238 kb)

## Abbreviations

Brz: Brassinazole, a kind of inhibitor of Brassinosteroids
CP: Cytokinin induced parthenocarpy
CPPU: N-(2-chloro-4-pyridyl)-N0-phenylurea, a diphenylurea-derived cytokinin
DEP: Differentially expressed protein
dpa: days post anthesis
EBR: Epi-Brassinosteroids
FDR: False discovery rate
iTRAQ: isobaric tags for relative and absolute quantitation
NP: NATURAL Parthenocarpy
QTL: Quantitative trait locus
STS: Silver thiosulphate
TIBA: 2,3,5-triodobenzoic acid, a kind of inhibitor of auxin

## References

[1] Pandolfini T (2009). Seedless fruit production by hormonal regulation of fruit set. Nutrients.

[2] Tiedjens VA (1928). The relation of environment to shape of fruit in Cucumis Sativus L. and its bearing on the genetic potentialities of the plants. J Agr Res.

[3] Denna DW (1973). Effects of genetic parthenocarpy and gynecious flowering habit on fruit production and growth of cucumber *Cucumis Sativus* L. J Am Soc Hortic Sci.

[4] Falavigna A, Soressi GP (1987). Influence of the pat-sha gene on plant and fruit traits in tomato (*L. esculentum* mill.). Modern trends in tomato genetics and breeding.

[5] Wang H, Jones B, Li Z (2005). The tomato aux/IAA transcription factor IAA9 is involved in fruit development and leaf morphogenesis. Plant Cell.

[6] Gorguet B, Eggink PM, Ocana J (2008). Mapping and characterization of novel parthenocarpy QTLs in tomato. Theor Appl Genet.

[7] Goetz M, Vivian-Smith A, Johnson SD (2006). AUXIN RESPONSE FACTOR8 is a negative regulator of fruit initiation in Arabidopsis. Plant Cell.

[8] Wittwer SH, Bukovac MJ, Sell HM (1957). Some effects of gibberellin on flowering and fruit setting. Plant Physiol.

[9] Martí C, Orzáez D, Ellul P (2007). Silencing of DELLA induces facultative parthenocarpy in tomato fruits. Plant J.

[10] Carrera E, Ruiz-Rivero O, Peres LEP (2012). Characterization of the procera tomato mutant shows novel functions of the SlDELLA protein in the control of flower morphology, cell division and expansion, and the auxin-signaling pathway during fruit-set and development. Plant Physiol.

[11] Mapelli S (1981). Changes in cytokinin in the fruits of parthenocarpic and normal tomatoes. Plant Sci Lett.

[12] Bohner J, Bangerth F (1988). Effects of fruit set sequence and defoliation on cell number, cell size and hormone levels of tomato fruits (*Lycopersicon esculentum* mill.) within a truss. Plant Growth Regul.

[13] Gillaspy G, Ben-David H, Gruissem W (1993). Fruits: a developmental perspective. Plant Cell.

[14] Srivastava A, Handa AK (2005). Hormonal regulation of tomato fruit development: a molecular perspective. J Plant Growth Regul.

[15] Trueman SJ (2010). Endogenous cytokinin levels during early fruit development of macadamia. Afr J Agric Res.

[16] Matsuo S, Kikuchi K, Fukuda M (2012). Roles and regulation of cytokinins in tomato fruit development. J Exp Bot.

[17] Ding J, Chen B, Xia X (2013). Cytokinin-induced parthenocarpic fruit development in tomato is partly dependent on enhanced gibberellin and auxin biosynthesis. PLoS One.

[18] Li Y, Yu JQ (2001). Photosynthesis and 14C-assimilate distribution as influenced by CPPU treatment on ovary. Acta Agric Nucleatae Sin.

[19] Vriezen WH, Feron R, Maretto F (2008). Changes in tomato ovary transcriptome demonstrate complex hormonal regulation of fruit set. New Phytol.

[20] Pascual L, Blanca JM, Cañizares J (2009). Transcriptomic analysis of tomato carpel development reveals alterations in ethylene and gibberellin synthesis during pat3/pat4 parthenocarpic fruit set. BMC Plant Biol.

[21] Martínez C, Manzano S, Megías Z (2013). Involvement of ethylene biosynthesis and signalling in fruit set and early fruit development in zucchini squash (*Cucurbita pepo* L.). BMC Plant Biol.

[22] Fos M, Nuez F, García-martínez JL (2000). The gene pat-2, which induces natural parthenocarpy, alters the gibberellin content in unpollinated tomato ovaries. Plant Physiol.

[23] Beraldi D, Picarella ME, Soressi GP (2004). Fine mapping of the parthenocarpic fruit (*pat*) mutation in tomato. Theor Appl Genet.

[24] Miyatake K, Saito T, Negoro S (2012). Development of selective markers linked to a major QTL for parthenocarpy in eggplant (*Solanum melongena* L.). Theor Appl Genet.

[25] Wu Z, Zhang T, Li L (2016). Identification of a stable major-effect QTL (Parth 2.1) controlling parthenocarpy in cucumber and associated candidate gene analysis via whole genome re-sequencing. BMC Plant Biol.

[26] Lietzow CD, Zhu H, Pandey S (2016). QTL mapping of parthenocarpic fruit set in north American processing cucumber. Theor Appl Genet.

[27] Smith O, Cochran HL (1935). Effect of temperature on pollen germination and tube growth in the tomato.

[28] Rylski I (1974). Effects of season on parthenocarpic and fertilized summer squash (*Cucumis pepo* L.). Exp Agric.

[29] Sun C, Li Y, Zhao W (2016). Integration of hormonal and nutritional cues orchestrates progressive corolla opening. Plant Physiol.

[30] Cholodny N (1928). Beiträge zur hormonalen Theorie von Tropismen. Planta.

[31] Went FW, Thimann KV (1937). Phytohormones.

[32] Rudich J, Baker LR, Sell HM. Parthenocarpy in *Cucumis sativus* L. as affected by genetic parthenocarpy, thermo-photoperiod, and femaleness. J Am Soc Hort Sci 1977;102(2):225-8.

[33] Matlob AN, Kelly WC (1975). Growth regulator activity and parthenocarpic fruit production in snake melon and cucumber grown at high temperature. J Am Soc Hortic Sci.

[34] Kim IS, Okubo H, Fujieda K (1992). Endogenous levels of IAA in relation to parthenocarpy in cucumber (*Cucumis sativus* L.). Sci Hortic.

[35] Huang S, Li R, Zhang Z (2009). The genome of the cucumber, *Cucumis sativus* L. Nat Genet.

[36] Hawthorn LR, Wellington R (1930). Geneva, a greenhouse cucumber that develops fruit without pollination. N Y State Agric Exp Station.

[37] Pike LM, Peterson CE (1969). Inheritance of parthenocarpy in the cucumber (*Cucumis sativus* L.). Euphytica.

[38] Kvasnikov BV, Rogova NT, Tarakanova SI, Ignatov SI (1970). Methods of breeding vegetable crops under the covered ground. Trudy Prikl Bot Genet Selek..

[39] Juldasheva L (1973). Inheritance of the tendency towards parthenocarpy in cucumbers. Byull Vsesoyuznogo Ordena Lenina Inst Rastenievodstva Imeni NI Vavilova.

[40] Meshcherov E, Juldasheva L (1974). Parthenocarpy in cucumber. Trudy Prikl Bot Genet Selek.

[41] Shawaf EI, Baker L (1981). Inheritance of parthenocarpic yield in gynoecious pickling cucumber for once-over mechanical harvest by diallel analysis of six gynoecious lines. J Am Soc Hortic Sci.

[42] Sun ZY, Lower RL, Staub JE (2006). Analysis of generation means and components of variance for parthenocarpy in cucumber (*Cucumis sativus* L.). Plant Breed.

[43] Yan LY, Lou LN, Li XL (2009). Evaluation of parthenocarpy in cucumber germplasm. Acta Hortic Sin.

[44] Robinson RW, Cantliffe DJ, Shannon S (1971). Morphactin-induced parthenocarpy in the cucumber. Science.

[45] Cantliffe D (1972). Parthenocarpy in the cucumber induced by some plant growth-regulating chemicals. Can J Plant Sci.

[46] Quebedeaux B, Beyer E (1972). Chemically-induced parthenocarpy cucumber by a new inhibitor of auxin transport. Hortscience.

[47] Elassar GJ, Patevitch D, Kedar N (1974). Induction of Parthenocarpic fruit development cucumber by growth regulators. Hortscience.

[48] Takeno K, Ise H, Minowa H (1992). Fruit growth induced by benzyladenine in Cucumis Sativus L.: influence of benzyladenine on cell division, cell enlargement and indole-3-acetic acid content. J Jpn Soc Hortic Sci.

[49] Yin Z, Malinowski R, Ziolkowska A (2006). The DefH9-iaaM-containing construct efficiently induces parthenocarpy in cucumber. Cell Mol Biol Lett.

[50] Ogawa Y, Inoue N, Aoki S (1989). Promotive effects of exogenous and endogenous gibberellins on the fruit development in *Cucumis sativus* L. J Jpn Soc Hortic Sci.

[51] Fu FQ, Mao WH, Shi K (2008). A role of brassinosteroids in early fruit development in cucumber. J Exp Bot.

[52] Hikosaka S, Sugiyama N (2015). Effects of exogenous plant growth regulators on yield, fruit growth, and concentration of endogenous hormones in Gynoecious Parthenocarpic cucumber (*Cucumis sativus* L.). Hortic J.

[53] Li J, Wu Z, Cui L (2014). Transcriptome comparison of global distinctive features between pollination and parthenocarpic fruit set reveals transcriptional phytohormone cross-talk in cucumber (*Cucumis sativus* L.). Plant Cell Physiol.

[54] Gustafson FG. Further studies on artificial parthenocarpy. Am J Bot. 1938a;25:237–44.

[55] Gustafson FG (1938). Induced parthenocarpy. Bot Gaz.

[56] Schwabe WW (1981). Hormones and parthenocarpic fruit set, a literature survey. Hortic Abstr.

[57] Vivian-Smith A, Koltunow AM (1999). Genetic analysis of growth-regulator-induced parthenocarpy in Arabidopsis. Plant Physiol.

[58] Spena A, Rotino GL, Bhajwani SS, Soh WY. Parthenocarpy. State of the art, Current trends in the embryology of Angiosperms. Kluwer Academic Publishers; 2001. p. 435-450.

[59] Marcelis LFM, Baan Hofman-Eijer LR (1993). Cell division and expansion in the cucumber fruit. J Hortic Sci.

[60] Huitrón MV, Diaz M, Diánez F (2007). Effect of 2, 4-D and CPPU on triploid watermelon production and quality. Hortscience.

[61] Bangerth F, Schröder M (1994). Strong synergistic effects of gibberellins with the synthetic cytokinin N-(2-chloro-4-pyridyl)-N-phenylurea on parthenocarpic fruit set and some other fruit characteristics of apple. Plant Growth Regul.

[62] Lewis DH, Burge GK, Hopping ME (1996). Cytokinins and fruit development in the kiwifruit (*Actinidia deliciosa*). II. Effects of reduced pollination and CPPU application. Physiol Plant.

[63] NeSmith DS (2002). Response of rabbiteye blueberry (*Vaccinium ashei* Reade) to the growth regulators CPPU and gibberellic acid. Hortscience.

[64] Boonkorkaew P, Hikosaka S, Sugiyama N (2008). Effect of pollination on cell division, cell enlargement, and endogenous hormones in fruit development in a gynoecious cucumber. Sci Hortic.

[65] Fang JB, Tian LL, Li SH (2000). Influence of CPPU on the sink and source of kiwifruit. Acta Horticulturae Sin.

[66] Zeng H, Yang W, Lu C (2016). Effect of CPPU on carbohydrate and endogenous hormone levels in young macadamia fruit. PLoS One.

[67] Ando K, Grumet R (2010). Transcriptional profiling of rapidly growing cucumber fruit by 454-pyrosequencing analysis. J Am Soc Hortic Sci.

[68] Ando K, Carr KM, Grumet R (2012). Transcriptome analyses of early cucumber fruit growth identifies distinct gene modules associated with phases of development. BMC Genomics.

[69] Thimm O, Bläsing O, Gibon Y (2004). MAPMAN: a user-driven tool to display genomics data sets onto diagrams of metabolic pathways and other biological processes. Plant J.

[70] Dreze M, Carvunis A-R, Charloteaux B (2011). Evidence for network evolution in an Arabidopsis interactome map. Science.

[71] Lü S, Zhao H, Des Marais DL (2012). Arabidopsis ECERIFERUM9 involvement in cuticle formation and maintenance of plant water status. Plant Physiol.

[72] Springer PS, Holding DR, Groover A (2000). The essential Mcm7 protein PROLIFERA is localized to the nucleus of dividing cells during the G (1) phase and is required maternally for early Arabidopsis development. Development.

[73] De Jong M, Wolters-Arts M, Feron R (2009). The Solanum Lycopersicum auxin response factor 7 (SlARF7) regulates auxin signaling during tomato fruit set and development. Plant J.

[74] Goetz M, Hooper LC, Johnson SD (2007). Expression of aberrant forms of AUXIN RESPONSE FACTOR8 stimulates parthenocarpy in Arabidopsis and tomato. Plant Physiol.

[75] Ren Z, Li Z, Miao Q (2011). The auxin receptor homologue in Solanum Lycopersicum stimulates tomato fruit set and leaf morphogenesis. J Exp Bot.

[76] Dharmasiri N, Dharmasiri S, Weijers D (2005). Plant development is regulated by a family of auxin receptor F box proteins. Dev Cell.

[77] Kepinski S, Leyser O (2005). The Arabidopsis F-box protein TIR1 is an auxin receptor. Nature.

[78] Guo H, Ecker JR (2003). Plant responses to ethylene gas are mediated by SCF EBF1/EBF2-dependent proteolysis of EIN3 transcription factor. Cell.

[79] Li J, Li Z, Tang L (2012). A conserved phosphorylation site regulates the transcriptional function of ETHYLENE-INSENSITIVE3-like1 in tomato. J Exp Bot.

[80] Key JL (1964). Ribonucleic acid and protein synthesis as essential processes for cell elongation. Plant Physiol.

[81] Faurobert M, Mihr C, Bertin N (2007). Major proteome variations associated with cherry tomato pericarp development and ripening. Plant Physiol.

[82] Weiler EW, Jourdan PS, Conrad W (1981). Levels of indole-3-acetic acid in intact and decapitated coleoptiles as determined by a specific and highly sensitive solid-phase enzyme immunoassay. Planta.

[83] Omar AA, Song WY, Grosser JW (2007). Introduction of Xa21, a Xanthomonas-resistance gene from rice, into ‘Hamlin’ sweet orange [C*itrus sinensis* (L.) Osbeck] using protoplast-GFP co-transformation or single plasmid transformation. J Hortic Sci Biotechnol.

[84] Bradford MM (1976). A rapid and sensitive method for the quantitation of microgram quantities of protein utilizing the principle of protein-dye binding. Anal Biochem.

[85] Wisniewski JR, Zougman A, Nagaraj N (2009). Universal sample preparation method for proteome analysis. Nat Methods.

[86] Wang Y, Zhang WZ, Song LF (2008). Transcriptome analyses show changes in gene expression to accompany pollen germination and tube growth in Arabidopsis. Plant Physiol.

[87] Rajjou L, Belghazi M, Huguet R (2006). Proteomic investigation of the effect of salicylic acid on Arabidopsis seed germination and establishment of early defense mechanisms. Plant Physiol.

[88] Menges M, Hennig L, Gruissem W (2002). Cell cycle-regulated gene expression in Arabidopsis. J Biol Chem.

[89] Hajduch M, Hearne LB, Miernyk JA (2010). Systems analysis of seed filling in Arabidopsis: using general linear modeling to assess concordance of transcript and protein expression. Plant Physiol.

[90] Hwang IS, Kim NH, Choi DS (2012). Overexpression of Xanthomonas campestris pv. Vesicatoria effector AvrBsT in Arabidopsis triggers plant cell death, disease and defense responses. Planta.

[91] Irshad M, Canut H, Borderies G (2008). A new picture of cell wall protein dynamics in elongating cells of Arabidopsis Thaliana: confirmed actors and newcomers. BMC Plant Biol.

