# Supplementary figures and images for: Proteomic insight into fruit set of cucumber (Cucumis sativus L.) suggests the cues of hormone-independent parthenocarpy
Source: BMC Genomics. 2017 Nov 22;18:896. doi: 10.1186/s12864-017-4290-5 (PMC5700656; doi:10.1186/s12864-017-4290-5)

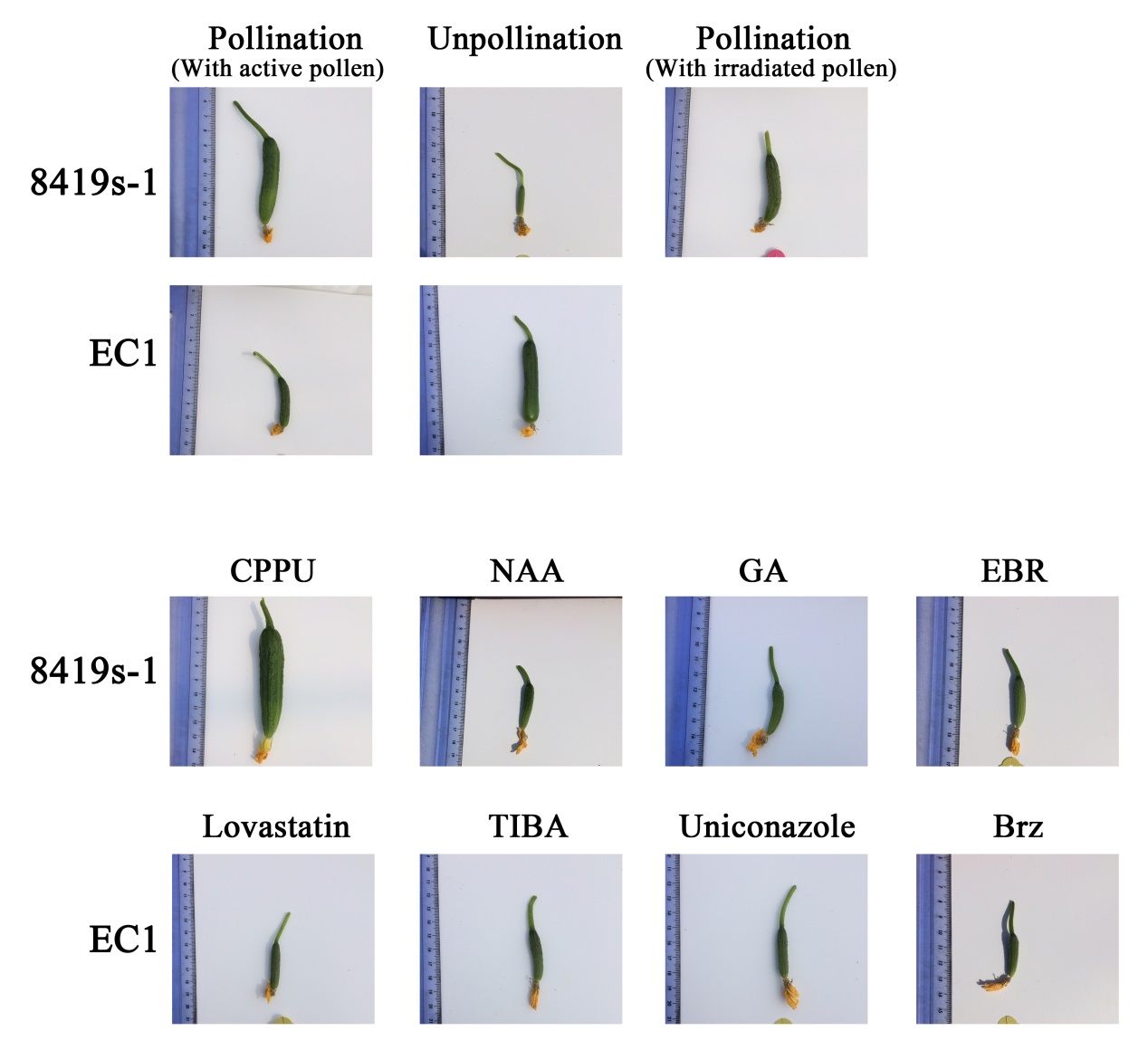


**Additional file 1: Figure S1.** The typical phenotypes of the treated ovaries of ‘EC1’ and ‘8419s-1’ at 4 dpa

Supplement: Supplementary file 1 — The typical phenotypes of the treated ovaries of ‘EC1’ and ‘8419 s-1’ at 4 dpa. (DOCX 196 kb) [file 12864_2017_4290_MOESM1_ESM.docx]
