# Supplementary material for: Proteomic insight into fruit set of cucumber (Cucumis sativus L.) suggests the cues of hormone-independent parthenocarpy
Source: BMC Genomics. 2017 Nov 22;18:896. doi: 10.1186/s12864-017-4290-5 (PMC5700656; doi:10.1186/s12864-017-4290-5)
Supplement: Supplementary file 3 — The analytical strategy of the iTRAQ based proteome analysis of cucumber fruits. Total proteins of each sample were extracted and labeled separately with tags (113 to 118). Proteomic analysis was conducted by iTRAQ. The differentially expressed proteins (DEPs) were identified by comparing proteomes of 0 and 2 dpa ovaries of each treatment. CK1 and CK2: Control sample 1 and Control sample 2; NP: natural parthenocarpic fruits of EC1; Unp: Unpollination fruits of 8419 s-1 (fruit abortion); P: pollination fruits of 8419 s-1; CP: Cytokinin induced parthenocarpic fruits of 8419 s-1. Bar = 20 mm. (DOCX 357 kb) [file 12864_2017_4290_MOESM3_ESM.docx]

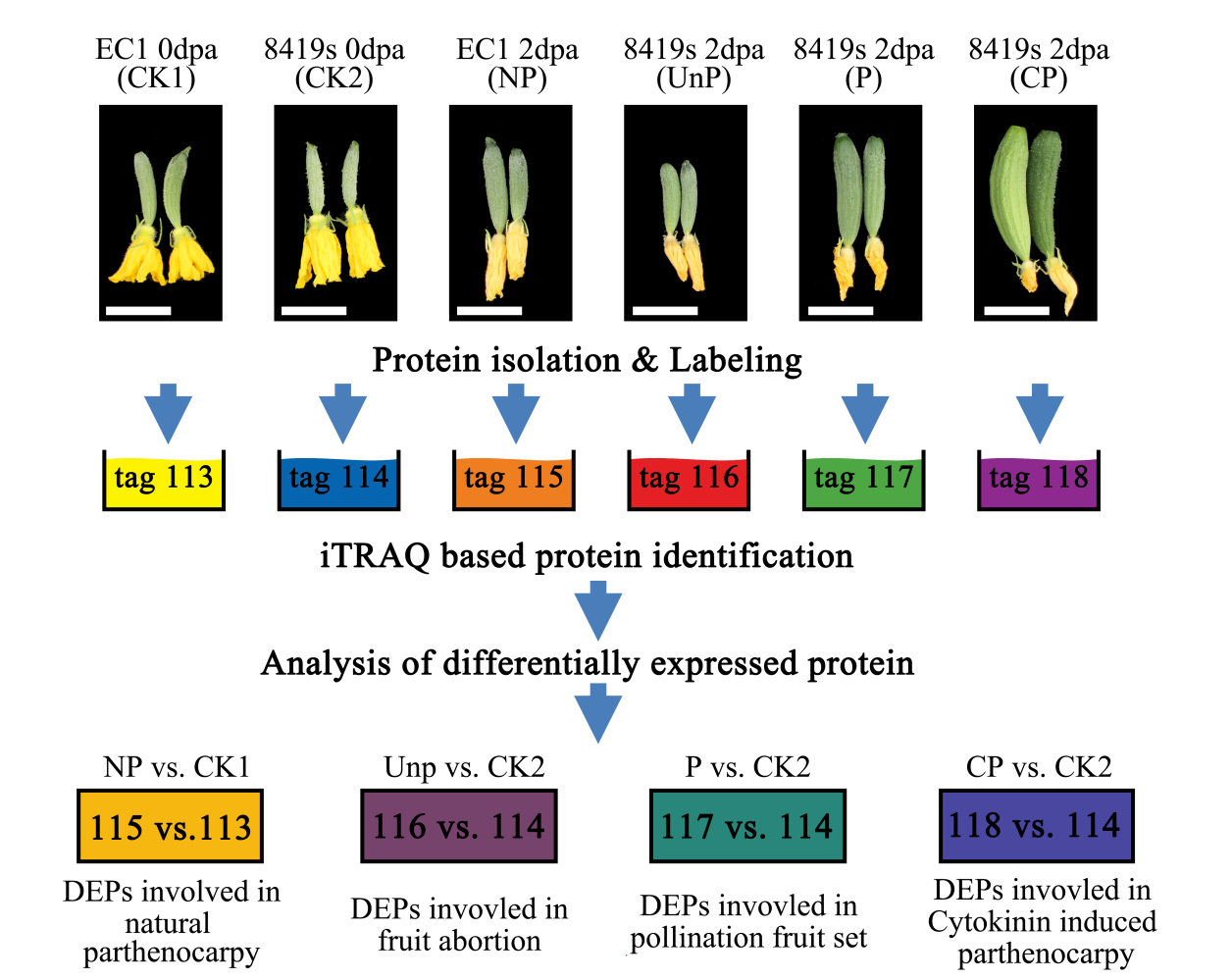


**Additional file 3: Figure S2.** The analytical strategy of the iTRAQ based proteome analysis of cucumber fruits

Total proteins of each sample were extracted and labeled separately with tags (113 to 118). Proteomic analysis was conducted by iTRAQ. The differentially expressed proteins (DEPs) were identified by comparing proteomes of 0dpa and 2dpa ovaries of each treatment.**CK1 and CK2:** Control sample1 and Control sample2; **NP:** natural parthenocarpic fruits of EC1; **Unp:** Unpollination fruits of 8419s-1 (fruit abortion); **P:** pollination fruits of 8419s-1; **CP:** Cytokinin induced parthenocarpic fruits of 8419s-1. Bar=20mm.
