# Supplementary material for: Proteomic insight into fruit set of cucumber (Cucumis sativus L.) suggests the cues of hormone-independent parthenocarpy
Source: BMC Genomics. 2017 Nov 22;18:896. doi: 10.1186/s12864-017-4290-5 (PMC5700656; doi:10.1186/s12864-017-4290-5)
Supplement: Supplementary file 4 — Statistics of differentially expressed proteins during different fruit developmental processes of cucumber. NP: natural parthenocarpic fruits of EC1; Unp: Unpollination fruits of 8419 s-1 (fruit abortion); P: pollination fruits of 8419 s-1; CP: Cytokinin induced parthenocarpic fruits of 8419 s-1. (DOCX 247 kb) [file 12864_2017_4290_MOESM4_ESM.docx]

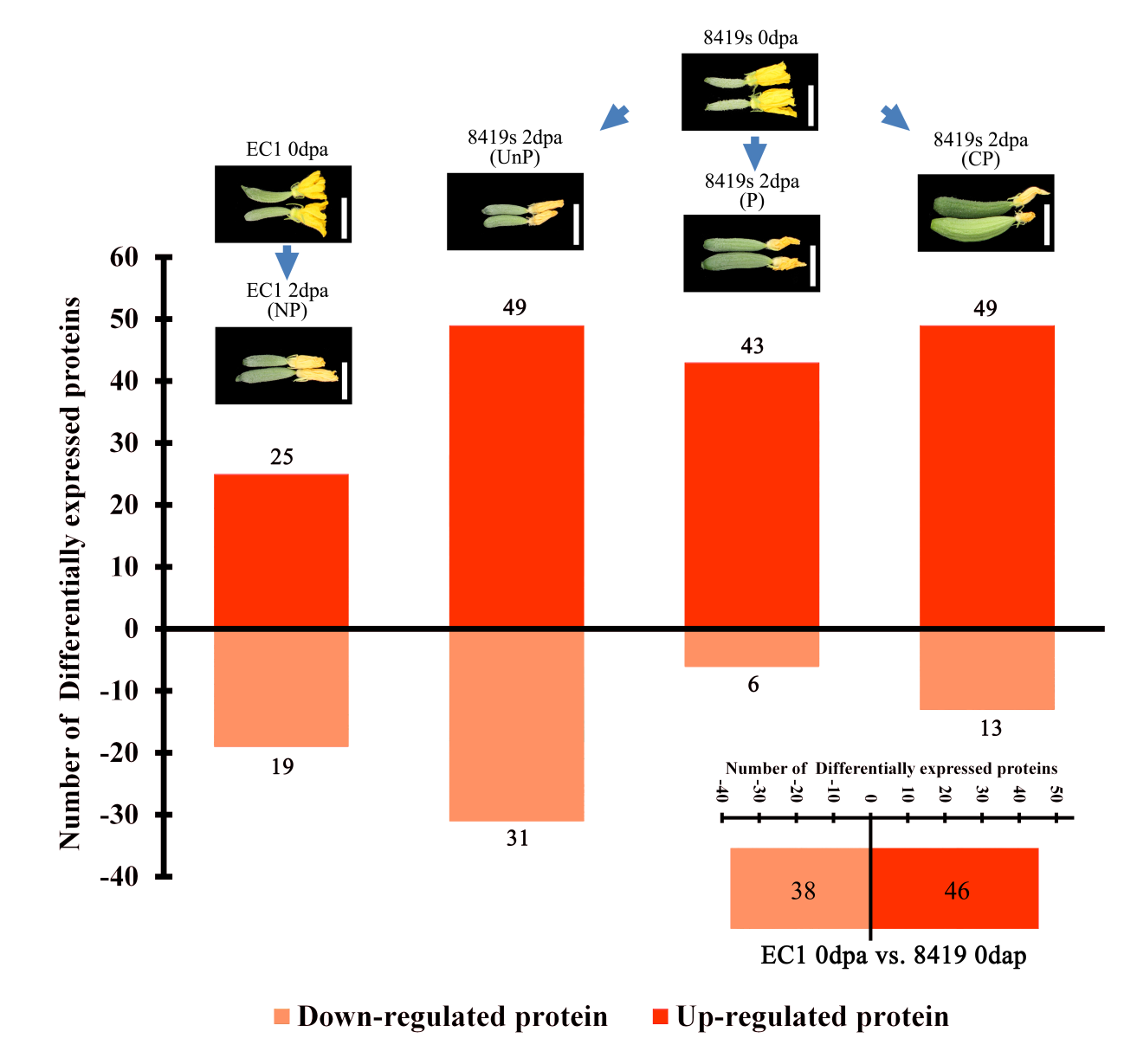


**Additional file 4: Figure S3.** Statistics of differentially expressed proteins during different fruit developmental processes of cucumber

**NP:** natural parthenocarpic fruits of EC1; **Unp:** Unpollination fruits of 8419s-1 (fruit abortion); **P:** pollination fruits of 8419s-1; **CP:** Cytokinin induced parthenocarpic fruits of 8419s-1.
