# Supplementary material for: Proteomic insight into fruit set of cucumber (Cucumis sativus L.) suggests the cues of hormone-independent parthenocarpy
Source: BMC Genomics. 2017 Nov 22;18:896. doi: 10.1186/s12864-017-4290-5 (PMC5700656; doi:10.1186/s12864-017-4290-5)
Supplement: Supplementary file 5 — Protein interactions occurred in different fruit developmental processes based on the predicted PPIs network. The interactions marked in red color means the interactions involved in Cytokinin induced parthenocarpy (A), natural parthenocarpy (B), pollination fruit set (C) and unpollination fruit abortion (D). (DOCX 349 kb) [file 12864_2017_4290_MOESM5_ESM.docx]

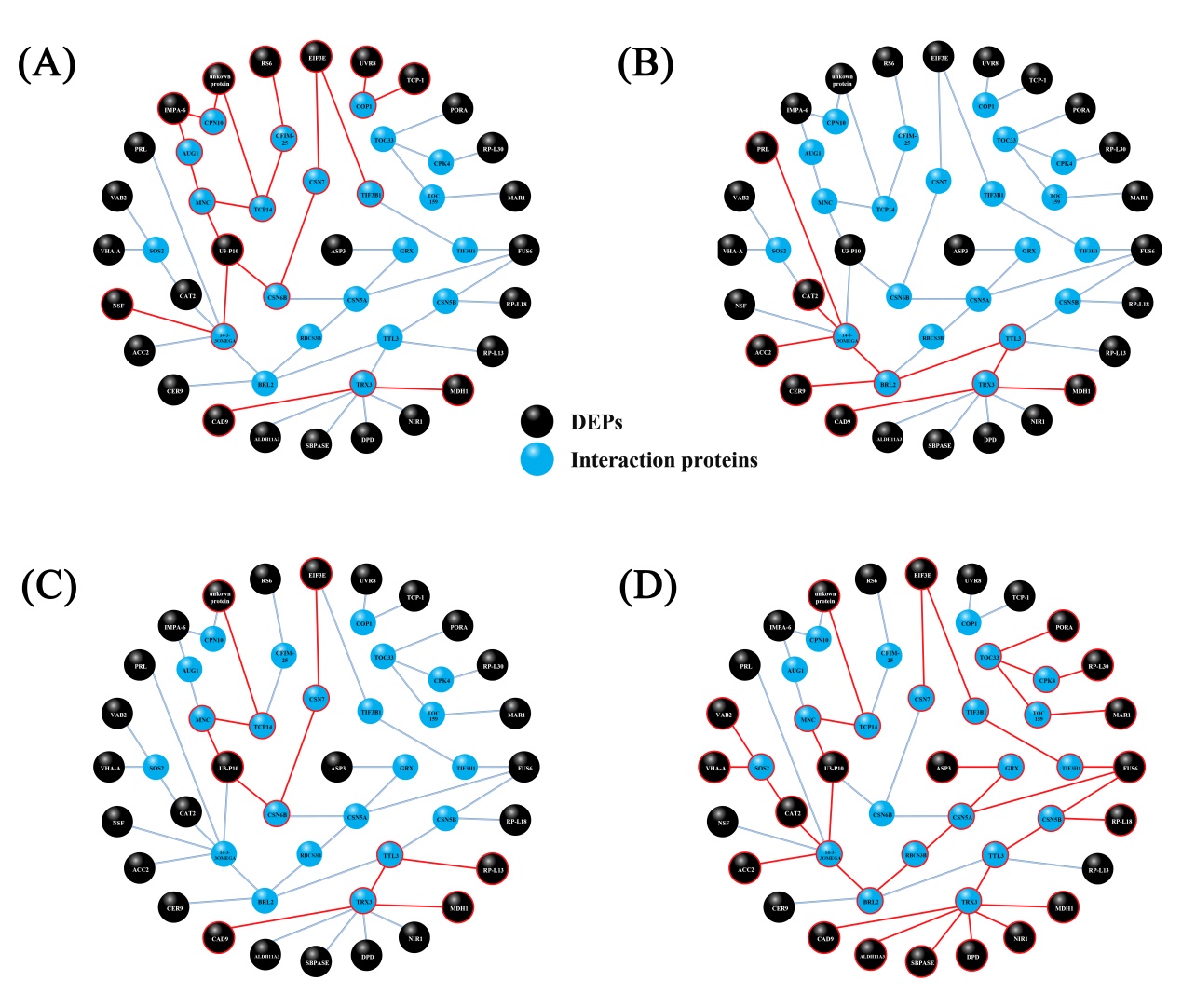


**Additional file 5: Figure S4.** Protein interactions occurred in different fruit developmental processes based on the predicted PPIs network

The interactions marked in red color means the interactions involved in Cytokinin induced parthenocarpy(A), natural parthenocarpy(B), pollination fruit set(C) and unpollination fruit abortion(D).
