# Supplementary material for: Proteomic insight into fruit set of cucumber (Cucumis sativus L.) suggests the cues of hormone-independent parthenocarpy
Source: BMC Genomics. 2017 Nov 22;18:896. doi: 10.1186/s12864-017-4290-5 (PMC5700656; doi:10.1186/s12864-017-4290-5)
Supplement: Supplementary file 6 — Band intensity analysis of western blotting by using the software ImageJ. The relative expression fold of each parthenocarpy specialized protein was calculated by the formula: (band intensity after hormone treatments/band intensity without hormone treatment)/(band intensity of beta-actin in hormone treated sample/band intensity of beta-actin in untreated sample). Each value represents the mean ± SE of three Western blotting replicates. (DOCX 238 kb) [file 12864_2017_4290_MOESM6_ESM.docx]

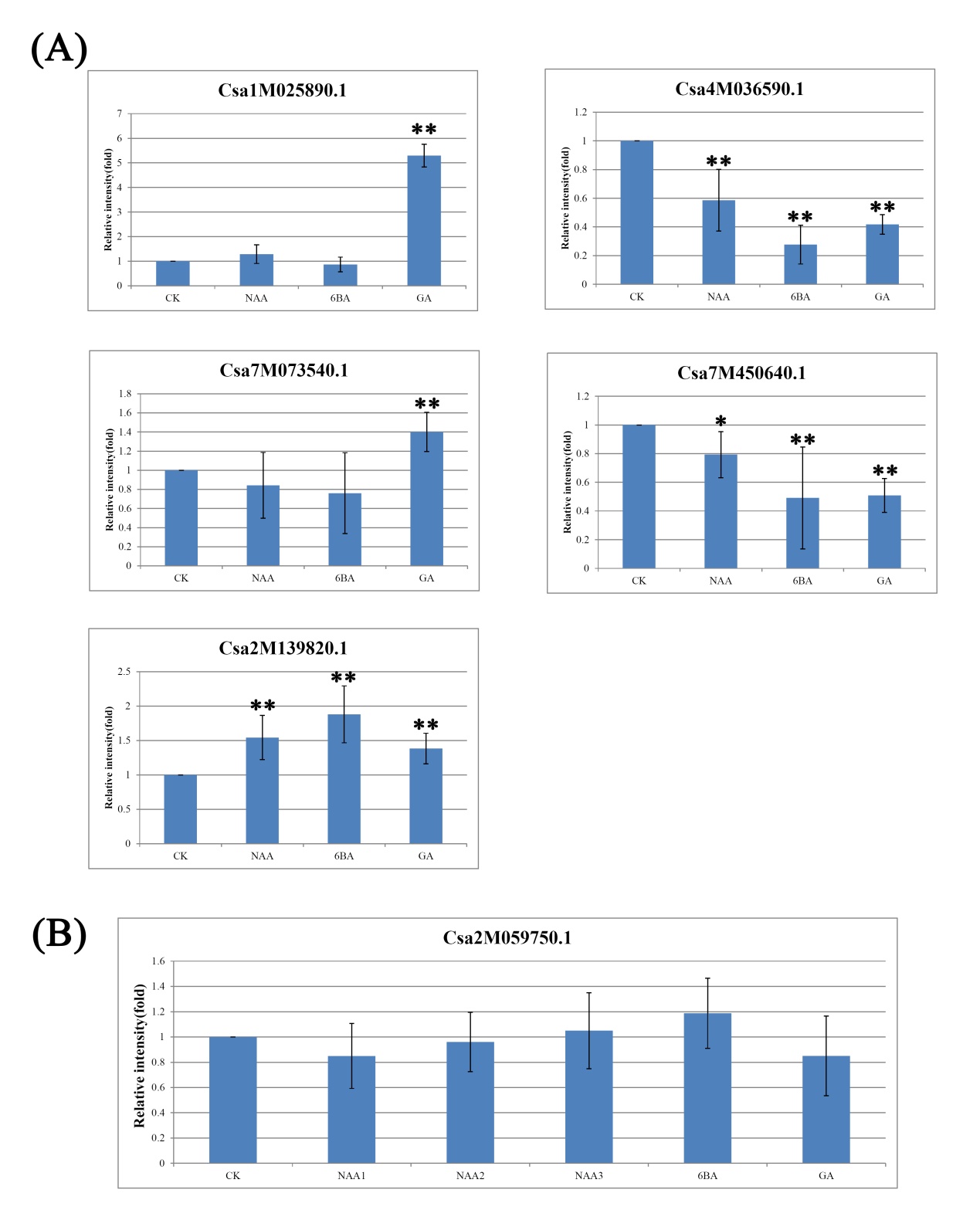


**Additional file 6: Figure S5.** Band intensity analysis of western blotting by using the software ImageJ

The relative expression fold of each parthenocarpy specialized protein was calculated by the formula: (band intensity after hormone treatments/band intensity without hormone treatment)/(band intensity of beta-actin in hormone treated sample/band intensity of beta-actin in untreated sample). Each value represents the mean ± SE of three Western blotting replicates.
